# Genomic prediction in contrast to a genome-wide association study in explaining heritable variation of complex growth traits in breeding populations of *Eucalyptus*

**DOI:** 10.1186/s12864-017-3920-2

**Published:** 2017-07-11

**Authors:** Bárbara S. F. Müller, Leandro G. Neves, Janeo E. de Almeida Filho, Márcio F. R. Resende, Patricio R. Muñoz, Paulo E. T. dos Santos, Estefano Paludzyszyn Filho, Matias Kirst, Dario Grattapaglia

**Affiliations:** 1 Cell Biology Department, Molecular Biology Program, Biological Sciences Institute, University of Brasília, Campus Darcy Ribeiro, Brasília, DF 70910-900 Brazil; 2 EMBRAPA Genetic Resources and Biotechnology, Estação Parque Biológico, Brasília, DF 70770-910 Brazil; 3 Forest Genomics Laboratory, School of Forest Resources and Conservation, University of Florida, Gainesville, FL 32611 USA; 4 RAPiD Genomics LLC, Gainesville, FL 32601 USA; 5 Agronomy Department, University of Florida, Gainesville, FL 32611 USA; 6 EMBRAPA Forestry, Estrada da Ribeira, Km 111, Colombo, PR 83411-000 Brazil

**Keywords:** Genomic selection, GWAS, SNP genotyping, Relatedness, Tree breeding, *Eucalyptus benthamii*, *E. pellita*

## Abstract

**Background:**

The advent of high-throughput genotyping technologies coupled to genomic prediction methods established a new paradigm to integrate genomics and breeding. We carried out whole-genome prediction and contrasted it to a genome-wide association study (GWAS) for growth traits in breeding populations of *Eucalyptus benthamii* (*n* =505) and *Eucalyptus pellita* (*n* =732). Both species are of increasing commercial interest for the development of germplasm adapted to environmental stresses.

**Results:**

Predictive ability reached 0.16 in *E. benthamii* and 0.44 in *E. pellita* for diameter growth. Predictive abilities using either Genomic BLUP or different Bayesian methods were similar, suggesting that growth adequately fits the infinitesimal model. Genomic prediction models using ~5000–10,000 SNPs provided predictive abilities equivalent to using all 13,787 and 19,506 SNPs genotyped in the *E. benthamii* and *E. pellita* populations, respectively. No difference was detected in predictive ability when different sets of SNPs were utilized, based on position (equidistantly genome-wide, inside genes, linkage disequilibrium pruned or on single chromosomes), as long as the total number of SNPs used was above ~5000. Predictive abilities obtained by removing relatedness between training and validation sets fell near zero for *E. benthamii* and were halved for *E. pellita*. These results corroborate the current view that relatedness is the main driver of genomic prediction, although some short-range historical linkage disequilibrium (LD) was likely captured for *E. pellita*. A GWAS identified only one significant association for volume growth in *E. pellita*, illustrating the fact that while genome-wide regression is able to account for large proportions of the heritability, very little or none of it is captured into significant associations using GWAS in breeding populations of the size evaluated in this study.

**Conclusions:**

This study provides further experimental data supporting positive prospects of using genome-wide data to capture large proportions of trait heritability and predict growth traits in trees with accuracies equal or better than those attainable by phenotypic selection. Additionally, our results document the superiority of the whole-genome regression approach in accounting for large proportions of the heritability of complex traits such as growth in contrast to the limited value of the local GWAS approach toward breeding applications in forest trees.

**Electronic supplementary material:**

The online version of this article (doi:10.1186/s12864-017-3920-2) contains supplementary material, which is available to authorized users.

## Background

Species of *Eucalyptus* are the most planted hardwood trees worldwide due to their multipurpose applications (e.g. pulp, paper, solid wood and bioenergy), superior growth, high adaptability and wood quality [1]. Amongst the 800 catalogued species of *Eucalyptus* L’Hér. (Myrtaceae), the “big nine” species within subgenus *Symphyomyrtus* account for over 95% of the world’s eucalypt plantations [2]. Within this group, *Eucalyptus grandis* Hill ex Maiden*, E. urophylla* S.T. Blake, and *E. camaldulensis* Dehnh are the most economically prominent ones in tropical regions, whereas *E. globulus* Labill and *E. nitens* H. Deane & Maiden are notable in temperate regions [1]. The extensive intra- and interspecific diversity and sexual compatibility across species of *Symphyomyrtus* has been a major advantage to breeders, as it allows rapid blending of gene pools that evolved separately under contrasting environmental pressures [3]. Nevertheless, there is still ample opportunities for expanding the use of some secondary species of *Symphyomyrtus* not included among the “big nine”, to develop uniquely adapted genetic material that combine rapid growth, good wood quality and adaptation to environmental stresses such as frost, heat and drought.

*Eucalyptus benthamii* Maiden & Cambage (Camden white gum), a species of restricted occurrence in its natural range in Australia [4], has showed great potential to expand eucalypt commercial plantations into subtropical regions subject to periodic frosts [5]. *Eucalyptus benthamii* planted as pure species or in hybrid combinations has received increasing attention in subtropical regions of southern Brazil and southeastern USA [6, 7]. Another species of marginal importance until recently, *Eucalyptus pellita* F. Mueller (large-fruited red mahogany), is highly suitable for growth in year-round humid lowland equatorial climates under high temperatures, showing a particularly high resistance to pathogens. *Eucalyptus pellita* is endemic to tropical regions in two disjoint natural forests, in southern New Guinea and in northern Australia [8]. It has shown fast growth in hybrid combination with *E. grandis* providing resistance to a number of fungal diseases [9].

Genomic selection (GS) was proposed by Meuwissen et al. [10], and has gained increasing interest among forest tree breeders. This predictive methodology provides an alternative approach to using marker-assisted selection (MAS) that relies on previously detected discrete quantitative trait loci (QTL) in bi-parental mapping and association genetics experiments. In forest trees, genomic prediction began to be addressed by simulation studies [11, 12] followed by experimental reports in *Pinus* [13] and *Eucalyptus* [14] demonstrating the positive prospects of this breeding method. Since then, a number of experimental genomic prediction studies have confirmed the potential of GS in conifer species, including *Pinus* [15–17] and *Picea* [18–21]. Recently, genomic prediction models were evaluated across generations in maritime pine (*Pinus pinaster*), [22, 23] demonstrating even more encouraging perspectives of this novel approach to accelerate breeding of forest trees.

Several parameters were shown to affect GS prediction accuracy in simulation studies, such as the number of QTLs controlling the trait, trait heritability, the size of the training population, number of markers and the effective population size (*N*_*e*_) of the target population [11]. If an adequate density of markers is provided for a given *N*_*e*_, it is expected that most QTL will be in LD with at least one marker and will be captured in predictive models. Consequently, high-throughput and low-cost genotyping platforms constitute an essential tool to apply GS. The reduction of the effective population size leads to increased relatedness between individuals and more extensive LD in the population. Markers fitted in a GS model will capture not only LD but also relatedness between individuals in the training and validation sets. An increase in prediction ability with enhanced relatedness among the training and validation sets was shown early on from simulation studies [24], and underscored in all recent reviews on the perspectives GS in plant and domestic animals breeding [25, 26]. Phenotypes of individuals closely related to the training population will be better predicted over distantly related individuals.

In this study, we report the development of genomic prediction models for growth traits in two breeding populations of *E. benthamii* (*n* =505) and *E. pellita* (*n* =732) using SNP data generated with the multi-species *Eucalyptus* EUChip60k SNP chip. Using a genomic relationship matrix (GRM) we compared the pedigree and genome-estimated breeding values and narrow-sense heritabilities in the two populations. Different Bayesian methods for predicting growth traits were compared. The impact of variable numbers of SNPs, different SNP sampling methods based on their position in the genome, and the impact of relatedness on genomic prediction were also evaluated. Finally, a genome-wide association analysis was carried out on the same datasets to evaluate what would be the ability to capture heritability and detect discrete associations for complex growth traits in an operational breeding population under selection.

## Methods

### Populations and phenotypic data

This study was carried out on progeny trials of populations of *E. benthamii* and *E. pellita* that are part of the breeding program of EMBRAPA (Brazilian Agricultural Research Corporation). The *E. benthamii* progeny trial was composed of 40 seed sources, being 36 open-pollinated (OP) half-sib families from wild Australian populations and four bulked seed sources (two from Australian populations, one from a first generation breeding population established in Colombo, PR, Brazil and one from a second-generation breeding population planted in Candói, PR, Brazil). The complete *E. benthamii* trial involved 2000 trees planted in May 2007 in Candói, in a randomized complete block design with 50 blocks in single-tree plots (one progeny individual per block for each one of the 40 seed sources). The experiment was thinned three times (removing 600 trees in March 2009, 700 in March 2010 and approximately 200 in December 2010) to eliminate trees with poor growth, malformed stems and damaged plants. The population underwent 25 heavy frosts recorded (temperature varying from −3.4 to −12.6 °C) in 58 months, between planting (May 2007) and field evaluation (February 2012) that killed or affected the growth of many trees which were therefore culled. For *E. benthamii* 508 trees were ultimately phenotyped at age 56 months for the following growth traits: Diameter at Breast Height (DBH, cm), Total Height (HT, m) and Wood Volume (WV, m^3^) (Table 1). The *E. pellita* breeding trial was composed of 24 OP maternal families derived from a second-generation clonal seed orchard located in Mareeba, Queensland, Australia, established with selections from four provenances in the areas of Kiriwo, Serisa and Keru in the Morehead district of the Western Province of Papua New Guinea. The experimental design was a randomized complete block design with 24 families and 40 blocks in single-tree plots (960 trees total) planted in February 2010 in Rio Verde, GO, Brazil. For *E. pellita* phenotypic evaluations were made at age 42 months (September 2013) for DBH, HT and WV (Table 1).

**Table 1:** General attributes of the breeding populations and trials studied

Phenotypic data	*E. benthamii*	*E. pellita*
Total number of trees in trial	2000	960
Total number of open pollinated (OP) families	40	24
Number of blocks	50	40
Number of individuals/OP family	10	32
Number of trees measured	508	747
Number of trees used in the analyses	505	732
Effective population size (*N*_*e*_) estimated from LD data	50	35
Age at phenotyping (*yr*)	4.6	3.5
Site	Candói, PR	Rio Verde, GO
Coordinates	25^o^43’00″S/ 52^o^11’00″W	25^o^36’00″S/ 52^o^03’00″W
Number of traits	3	3

### Genotyping and filtering

A total of 552 *E. benthamii* trees and 771 *E. pellita* trees were genotyped using the *Eucalyptus* Illumina Infinium EUChip60K [27]. The genotypic data were filtered to remove SNPs with call rate (CR) ≤ 90% and monomorphic SNPs, therefore keeping all SNPs with Minimum Allele Frequency (MAF) > 0 in the analysis. Because trees were genotyped before the final field measurements, some genotyped trees died, so that ultimately 505 individuals of *E. benthamii* and 732 of *E. pellita* had full genotypic and phenotypic data for further analyses. An alternative SNP dataset was also generated by keeping only SNPs MAF ≥0.05. With the objective of evaluating the effect of LD-pruning on predictions, polymorphic SNPs (CR ≥ 90% and MAF > 0 or MAF ≥ 0.05) were pruned based on pairwise linkage disequilibrium (LD) estimates using PLINK v1.9 [28], to generate a pruned subset of SNPs that are in approximate linkage equilibrium (LE). The LD based SNP pruning method was applied with a window size of 100 Kbp, shifting the window by one SNP at the end of each step and removing one SNP from a pair of SNPs if LD was greater than 0.2 (plink command: --indep-pairwise 100 kb 1 0.2).

### Effective population size estimation, population structure and LD analyses

Effective population size (*N*_*e*_) was estimated based on the linkage disequilibrium (LD*N*_*e*_) method implemented in NeEstimator v2.01 [29] for each species. A random mating model and MAF < 0.05 was used for excluding rare alleles in LD*N*_*e*._ Confidence intervals for these estimates were obtained using the parametric method in NeEstimator, where the number of independent alleles is used as the degree of freedom in a chi-square distribution. The genetic structure for both eucalypt populations estimated based on a Bayesian clustering method was determined with STRUCTURE v2.2.4 [30] using only the LD-pruned SNPs set. The individual structures were classified in *K* clusters according to genetic similarity. The admixture model was applied, with correlated allelic frequencies, using no previous population information. The number of tested clusters (*K*) ranged from 1 to 10, and each *K* was replicated 10 times. The burn-in period and the number of Markov Chain Monte Carlo (MCMC) replications were 100,000 and 200,000, respectively. The number of genetic groups was determined based on the criteria proposed by Evanno et al. [31] using the program STRUCTURE HARVESTER v0.6.93 [32]. The software CLUMPP v1.1.2 [33] was used to find consensus among the 10 most probable *K* interactions. Principal component analysis (PCA) was performed using SNPRelate R package [34], with only the LD-pruned SNPs set. Analyses of linkage disequilibrium were performed using LDcorSV [35]. Pairwise estimates of LD were calculated by the classical measure of the squared correlation of allele frequencies at diallelic loci (*r* ^*2*^), as well as correcting for bias due to relatedness and population structure (*r* ^*2*^ *VS*), and adjusting it independently for relatedness (*r* ^*2*^ *V*) and for population structure (*r* ^*2*^ *S*). To estimate the adjusted LD, the genomic relationship matrix (GRM) was computed using the Powell method [36] implemented in R. The population structure results were based on the most probable value of *K* (*K = 2*). The LD decay of *r* ^*2*^ with distance in Kbp was fitted by a nonlinear regression model between adjacent sites using the R script by Marroni et al. [37]. To visualize patterns of LD decay in the two eucalypts species, all the LD estimates (*r* ^*2*^, *r* ^*2*^ *V*, *r* ^*2*^ *S*, *r* ^*2*^ *VS*) were plotted up to a 100 Kbp distance.

### Genomic and pedigree-based breeding value predictions

Prediction of breeding values by best linear unbiased prediction (BLUP) [38] based on pedigree information (ABLUP) was calculated using the expected genetic relationship between individuals. For the genomic estimated breeding values the individual SNPs had their effects estimated by adjusting all the allelic effects simultaneously using Genomic BLUP (GBLUP) frequentist [39]. A 10-fold cross-validation approach was used, defined as a random subsampling partitioning of the data for each trait into two subsets. The first subset with 90% of the individuals was used as a training population to estimate the marker effects. The second subset with the remaining 10% was used as validation population, and had their phenotypes predicted based on the marker effects estimated in the training population. This process was repeated 10 times, randomly selecting in each fold a different set of samples as the validation population, until all individuals had their phenotypes predicted and validated. Analyses of each trait were carried out using the package rrBLUP [40] with the following mixed linear model:

$$ y= Xb+ Za+ e $$

where *y* is the phenotypic measure of the trait being analyzed; *X* and *Z* are incidence matrices for the vectors for parameters *b* and *a*, respectively; *b* is a vector of fixed block effects; *a* is a vector of random additive effects and *e* is the random residual effect. The variance structure of the model for pedigree-estimated breeding values or simply estimated breeding values (EBVs) was calculated with $a\sim N\left(0, A{\sigma}_a^2\right)$ and the genomic-estimated breeding values (GEBVs) with $a\sim N\left(0, G{\sigma}_a^2\right)$; where *A* is a matrix of additive genetic relationships among individuals and *G* is a GRM estimated using the method proposed by VanRaden [39]. The predictive ability (*r*_*gy*_) was estimated as the correlation between the observed and the genomic-estimated breeding values (*r*(*y*, *GEBV*)). The narrow-sense heritability (*h* ^2^) was calculated as the ratio of the additive variance ${\sigma}_a^2$ to the phenotypic variance ${\sigma}_y^2$ $\left({h}^2={\sigma}_a^2/{\sigma}_y^{2\ }\right)$.

### Bayesian methods

The SNP effects were estimated using five different Bayesian genome-wide regression models, namely Bayesian Ridge-Regression (BRR), Bayes A, Bayes B, Bayes Cπ and Bayesian Lasso (BL) as implemented in the BGLR package [41]. For these methods the genotypic information was fitted using the following base model:

$$ y= Xb+ Zm+ e $$

where *y* is the vector of observations representing the trait of interest; *b* is a vector with intercept and fixed block effects; *m* is a vector of random markers effects (*m* = [*m*_1_  …  *m*_*k*_]^*T*^); *X* and *Z* are incidence matrices for the vectors for parameters *b* and *m*, respectively; *e* is a vector of the random error effects. The *Z* matrix takes values 2, 1 or 0 if the genotype of the *i* ^*th*^ marker is AA, Aa and aa, respectively, where a is the least frequent allele. Missing genotypes were replaced by the mean of the genotype for the given SNP. In all Bayesian models it was assumed that:

$$ y\left| b, m,{\sigma}_e^2\sim N\left( Xb+ Zm, I{\sigma}_e^2\right)\right. $$

$$ b\sim N\left(0,{10}^6 I\right) $$

$$ e\left|{\sigma}_e^2\sim N\left(0, I{\sigma}_e^2\right)\right. $$

$$ {\sigma}_e^2\left|{S}_e,{\nu}_e\sim {\chi}^{-2}\left({\nu}_e,{S}_e\right)\right. $$

The assumptions of the *m* vector depend on the prior adopted. The respective priors used in the linear regression coefficients for each model are described in Additional file 1. To estimate the parameters of the models a total 200,000 iterations of MCMC were used with a burn-in period of 50,000 cycles and every fifth sample was kept. For all these models, a 10-fold cross-validation approach was applied as described previously.

### Genomic predictions using selected SNPs subsets

The Bayesian Ridge-Regression (BRR) model was fitted using different subsets of SNPs of various sizes and selected using different criteria as described below. Initially a random sampling of SNPs stratified by chromosome was tested using (i) a cumulative approach, such that from the smallest subset of SNPs tested, additional ones were added to the previous set and (ii) a non-cumulative fashion, where different final sets of SNPs were randomly selected from all available SNPs. Next, variable positions of SNPs were tested, including: (iii) evenly spaced SNPs across the genome; (iv) only SNPs within gene models annotated in the *Eucalyptus* reference genome [42]; (v) SNPs based on LD-pruning and (vi) SNPs from individual chromosomes. For each subset we estimated the predictive ability and genomic heritability. First, we evaluated models using different SNP subsets (from all 13,787 and 19,506 SNPs available for *E. benthamii* and *E. pellita* respectively, down to 2000 in smaller increments of 1000 SNPs, 1500, 1250, 1000, 750, 500, 300, 250, 200, 150 and 100 SNPs) with either a cumulative (i) or non-cumulative (ii) sampling of SNPs. For each number of SNPs and sampling strategy, ten replicates were performed. The evenly spaced SNPs subsets (iii) were created using different target windows sizes, with 1 SNPs every 10, 50, 100, 250, 500 Kbp and 1 Mbp, resulting in variable average distances between SNPs (Additional file 2: Table S1). For the within-gene SNP subset (iv), all SNPs located within annotated gene models (genic regions) and SNPs located outside of annotated gene models (intergenic regions) in the *Eucalyptus* genome were evaluated. To create the subsets of SNPs selected based on LD pruning (v), SNPs in approximate LE (*r* ^*2*^ ≤ 0.2) with each other were chosen using PLINK v1.9 [28]. Finally, in the chromosome-specific SNP subsets (vi) the prediction models were fitted independently using only SNPs on each chromosome separately.

### Genomic prediction controlling for relatedness between training and validation sets

To assess the relative impact of relatedness versus historical LD on the predictive ability, BRR prediction models were fitted minimizing relatedness between training and validation populations. Individuals were split into training and validation sets based on a Principal Component Analysis (PCA) or STRUCTURE analysis (*K = 2*). In *E. benthamii*, 21 outlier individuals were removed and the remaining individuals were split into two subpopulations based on maximum genetic distance, one with 310 trees and the other with 174. For *E. pellita*, 26 outliers were excluded and the remaining 706 individuals were split into two subpopulations with 192 and 514 trees. As a control, a 10-fold cross-validation in each direction, with the same numbers of individuals used in the split populations, was carried out by random allocation of the individuals to training and validation sets.

### Genome-wide association analysis

A mixed linear model association (MLMA) analysis was performed using the GCTA software [43]. This association analysis was fitted using the following base model:

$$ y= Xb+ g+ e $$

where *y* is the phenotype; *b* is a vector of fixed effects including intercept, block, population structure and SNPs to be tested for association; *X* is the incidence matrix for the vectors for the parameters *b*; *g* is the polygenic effect (random effect) captured by the GRM calculated using all SNPs and *e* is the random residual effect. The covariate computed for population structure was based on the fact that the population had two subpopulations (*K* = 2). The variance structure of the MLMA model were $g\sim N\left(0, G{\sigma}_g^2\right)$; $e\sim N\left(0, I{\sigma}_e^2\right)$; cov(*g*, *e* ^′^) = 0, where *G* is the GRM between individuals calculated as described earlier [44] and *I* is the identity matrix. For comparisons with the MLMA model, we also performed a linear model based association (LMA) analysis fitting each SNP independently. This single-SNP association analysis was carried out using PLINK [28] using a similar model as MLMA, except for the exclusion of the polygenic effect (*g*). The Bonferroni procedure was implemented to control for type I error at α = 0.05 and the Benjamini & Hochberg procedure [45] was used to control for false discovery at a rate FDR = 5%. The quantile-quantile (Q-Q) and Manhattan plots were generated using the qqman R package [46].

## Results

### SNP genotyping

Of the 60,904 SNPs in the EUChip60K, 50,303 (82.6%) and 49,518 (81.3%) were genotyped for *E. benthamii* and *E. pellita* respectively (Additional file 2: Figure S1A), by using the phylogenetically appropriate SNP clustering file for SNP calling [27], and filtering for SNPs with CR ≥ 90%. After selecting polymorphic SNPs (MAF > 0) 13,787 and 19,506 SNPs were retained for further analyses with a final rate of missing data of 1.4% and 0.8% for *E. benthamii* and *E. pellita*, respectively. An alternative SNP dataset was also used by filtering out SNPs with MAF < 0.05 to investigate whether removing lower frequency SNPs had an impact on genomic predictions. A total of 7563 SNPs for *E. benthamii* and 12,483 SNPs for *E. pellita* were retained for this alternative set.

### Linkage disequilibrium and estimated effective population sizes

**Fig. 1:**
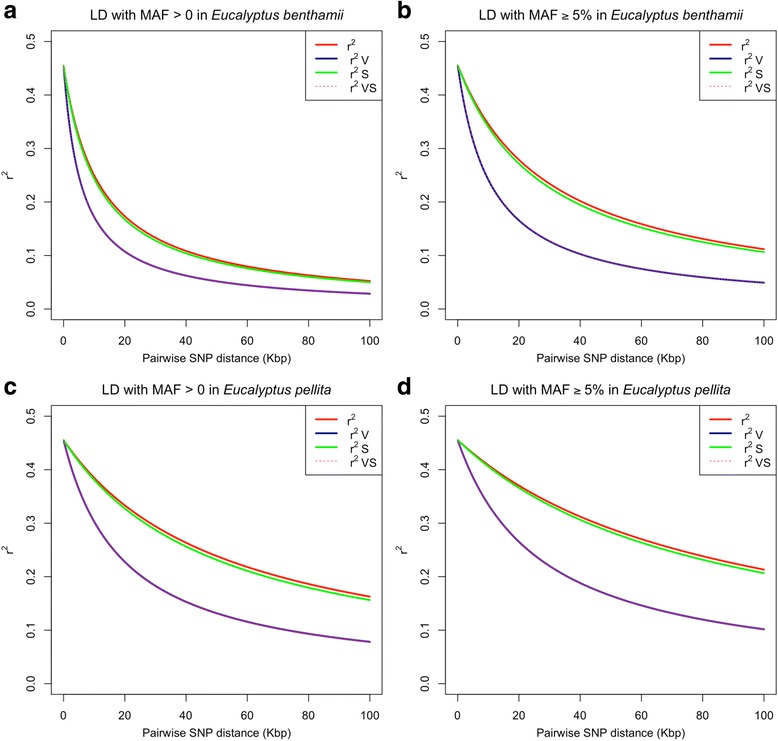
Genome-wide pattern of Linkage Disequilibrium (LD) decay up to 100 Kbp pairwise SNP distances. Decay curves of the classical measure of the squared correlation of allele frequencies at diallelic loci (*r* ^*2*^), adjusted for population structure (*r* ^*2*^ *S*) and relatedness (*r* ^*2*^ *V*), and adjusted for both (*r* ^*2*^ *VS*). **a** Plot with SNPs filtered using MAF > 0 and **b** MAF ≥ 5% in *E. benthamii*. **c** Plot with SNPs filtered using MAF > 0 and **d** MAF ≥ 0.05 in *E. pellita*

Linkage disequilibrium (*r* ^*2*^) was calculated for all pairwise physical distances among all the polymorphic SNPs (MAF > 0) on each chromosome separately. The average, genome-wide LD for pair of SNPs within a 100 Kbp distance from each other was 0.141 and 0.271 for *E. benthamii* and *E. pellita*, respectively. When correcting the LD for biases due to relatedness and population structure (*r* ^*2*^ *VS*), the average estimates were reduced to 0.096 and 0.178 (Additional file 2: Table S2). The genome-wide LD decayed to an *r* ^*2*^ below 0.2 within 15.6 Kb and 70.6 Kb (red line), while *r* ^*2*^ *VS* showed a slightly faster decay within 7.7 and 25.5 Kb (pink dots) for *E. benthamii* and *E. pellita*, respectively (Fig. 1a and c). Linkage disequilibrium decayed to <0.2 for *r* ^*2*^ *S* (correcting for population structure) within 14.7 and 66.2 Kb (green line), while *r* ^*2*^ *V* (correcting for relatedness) showed a slightly faster decay within 7.7 and 25.6 Kb (blue line), very similar to *r* ^*2*^ *VS* for *E. benthamii* and *E. pellita*, respectively (Fig. 1a and c, Additional file 2: Table S2). The faster LD decay for *r* ^*2*^ *V* or *r* ^*2*^ *VS* confirms the strong effect of genetic relationship in these breeding populations. Slightly different patterns of LD decay were observed when including the SNPs with MAF < 0.05 (Fig. 1a and c, MAF > 0) or excluding those (Fig. 1b and d). Datasets without the SNPs with MAF < 0.05 showed a slightly higher pairwise *r* ^*2*^, with corrected LD (*r* ^*2*^ *VS*) decaying to *r* ^*2*^ = 0.2 at 14.5 Kb in *E. benthamii* and 35.8 Kb in *E. pellita* (Fig. 1b and d, Additional file 2: Table S2). Estimated effective populations sizes based on LD data were *N*_*e*_ = 50 and *N*_*e*_ = 35 for *E. benthamii* and *E. pellita*, respectively (Table 1).

### Genomic and pedigree-estimated heritabilities

For *E. benthamii* the pedigree-based narrow-sense heritabilities (*h* ^*2*^) estimated for DBH and WV were 0.326 and 0.297, and considerably lower for HT (0.088). Estimates of genomic heritabilities varied depending on the method used, with GBLUP and BL yielding considerably lower heritabilities than the pedigree-based ones and those obtained using other Bayesian methods (Table 2). When using Bayes B and BRR, heritabilities were higher (0.155 and 0.190). Estimates of variance components are reported in Additional file 3. In *E. benthamii*, the variance components had similar estimates with all methods used. The pedigree-based narrow-sense heritabilities estimated for *E. pellita* were zero for DBH and WV, and nearly zero for HT (0.019), while the genomic estimated heritabilities based on SNP data were considerably higher (e.g. 0.414–0.527 for DBH using the different methods) (Table 2). This unexpected result strongly suggests that the informed pedigrees for the *E. pellita* population do not match the true relationships that the SNP data correctly recovered. Differently from *E. benthamii*, in *E. pellita* the genomic heritabilities had similar estimates for all methods used. Average heritabilities for *E. pellita* considering all genomic methods (~0.47 for DBH; ~0.29 for HT; ~0.44 for WV) were higher for all traits, compared to those estimated for *E. benthamii* (~0.23 for DBH; ~0.09 for HT; ~0.20 for WV). Heritabilities estimated including or not lower frequency SNPs (MAF < 0.05) in the genomic relationship matrix were equivalent for both species, varying within the standard error of the estimates (Table 2). Genomic heritabilites captured large proportions of the pedigree-based heritability in *E. benthamii*. The Bayesian methods on average captured 73% and 69% of the pedigree-heritability for DBH and WV, respectively. For HT, however, genomic heritabilities varied considerably depending on the method, at times surpassing the pedigree-based estimate. No assessment was possible for *E. pellita* due to the inconsistency of the pedigree data that provided no valid estimate of pedigree-based heritability.

**Table 2:** Estimates of narrow-sense heritabilities (*h* ^*2*^) and predictive abilities (*r*_*gy*_), obtained using pedigree data (ABLUP) and genomic data (several methods), for the *E. benthamii* and *E. pellita* breeding populations

Method	Filter	*E. benthamii*						*E. pellita*					
		DBH		HT		WV		DBH		HT		WV	
		*h* ^*2*^	*r*_*gy*_ (SE)	*h* ^*2*^	*r*_*gy*_ (SE)	*h* ^*2*^	*r*_*gy*_ (SE)	*h* ^*2*^	*r*_*gy*_ (SE)	*h* ^*2*^	*r*_*gy*_ (SE)	*h* ^*2*^	*r*_*gy*_ (SE)
ABLUP		0.326 (NA)	0.148 (0.045)	0.088 (NA)	0.090 (0.033)	0.297 (NA)	0.142 (0.039)	0.000 (NA)	- 0.030 (0.028)	0.019 (NA)	0.040 (0.028)	0.000 (NA)	- 0.009 (0.026)
GBLUP	MAF > 0	0.181 (NA)	0.157 (0.044)	0.000 (NA)	0.006 (0.044)	0.147 (NA)	0.141 (0.041)	0.466 (NA)	0.439 (0.019)	0.260 (NA)	0.342 (0.042)	0.424 (NA)	0.424 (0.028)
Bayes A		0.202 (0.017)	0.160 (0.045)	0.058 (0.016)	0.010 (0.040)	0.165 (0.020)	0.141 (0.041)	0.465 (0.008)	0.440 (0.019)	0.280 (0.011)	0.342 (0.042)	0.428 (0.008)	0.424 (0.028)
Bayes B		0.287 (0.032)	0.166 (0.045)	0.155 (0.052)	0.003 (0.041)	0.284 (0.028)	0.146 (0.038)	0.527 (0.020)	0.439 (0.019)	0.341 (0.017)	0.342 (0.042)	0.517 (0.025)	0.425 (0.028)
Bayes Cπ		0.267 (0.017)	0.158 (0.044)	0.109 (0.007)	0.016 (0.039)	0.237 (0.014)	0.148 (0.039)	0.480 (0.007)	0.439 (0.019)	0.303 (0.009)	0.342 (0.042)	0.453 (0.007)	0.423 (0.028)
BL		0.133 (0.019)	0.155 (0.045)	0.044 (0.004)	0.010 (0.042)	0.103 (0.011)	0.140 (0.041)	0.414 (0.021)	0.434 (0.021)	0.242 (0.014)	0.338 (0.043)	0.406 (0.007)	0.424 (0.028)
BRR		0.267 (0.008)	0.162 (0.044)	0.190 (0.005)	0.022 (0.036)	0.243 (0.008)	0.146 (0.039)	0.455 (0.005)	0.441 (0.019)	0.283 (0.008)	0.342 (0.042)	0.418 (0.005)	0.425 (0.028)
GBLUP	MAF ≥ 0.05	0.179 (NA)	0.153 (0.044)	0.000 (NA)	0.009 (0.044)	0.144 (NA)	0.138 (0.041)	0.457 (NA)	0.437 (0.020)	0.254 (NA)	0.340 (0.042)	0.419 (NA)	0.422 (0.028)
Bayes A		0.214 (0.013)	0.158 (0.045)	0.073 (0.008)	0.020 (0.040)	0.190 (0.017)	0.144 (0.041)	0.463 (0.007)	0.438 (0.020)	0.279 (0.008)	0.340 (0.042)	0.437 (0.005)	0.422 (0.028)
Bayes B		0.354 (0.041)	0.162 (0.045)	0.110 (0.016)	0.019 (0.040)	0.269 (0.029)	0.146 (0.040)	0.551 (0.020)	0.438 (0.019)	0.393 (0.036)	0.339 (0.042)	0.501 (0.010)	0.423 (0.028)
Bayes Cπ		0.259 (0.011)	0.157 (0.046)	0.116 (0.006)	0.020 (0.039)	0.232 (0.008)	0.143 (0.040)	0.485 (0.005)	0.437 (0.020)	0.300 (0.008)	0.340 (0.042)	0.449 (0.007)	0.423 (0.028)
BL		0.143 (0.023)	0.153 (0.043)	0.045 (0.003)	0.020 (0.041)	0.101 (0.009)	0.134 (0.041)	0.408 (0.009)	0.427 (0.023)	0.244 (0.010)	0.339 (0.041)	0.403 (0.006)	0.422 (0.029)
BRR		0.260 (0.007)	0.158 (0.044)	0.184 (0.004)	0.025 (0.036)	0.239 (0.006)	0.143 (0.040)	0.443 (0.005)	0.437 (0.020)	0.280 (0.008)	0.341 (0.042)	0.415 (0.006)	0.422 (0.028)

NA - The standard error of the heritability could not be estimated using rrBLUP

*Pedigree BLUP* (ABLUP, Pedigree Best Linear Unbiased Predictor), *Genomic BLUP* (GBLUP, Genomic Best Linear Unbiased Predictor), *BL* (Bayesian Lasso), *BRR* (Bayesian Ridge-Regression), *MAF* (Minimum Allele Frequency), *DBH, cm* (Diameter at Breast Height), *HT, m* (Total Height) and *WV, m* ^*3*^ (Wood Volume), *SE* (Standard Error)

### Genomic predictions

Consistent with expectations, predictive abilities (*r*_*gy*_) followed the same trend as the estimated genomic heritabilities (Table 2). Predictive abilities estimated using different Bayesian methods produced equivalent estimates to those obtained using GBLUP and pedigree-based. For the *E. benthamii* population both pedigree and genomic predictive abilities were generally low, averaging 0.16 for DBH, 0.14 for WV and close to zero for HT across all methods. For *E. pellita*, genomic predictive abilities were considerably higher, averaging 0.44 for DBH, 0.34 for HT and 0.42 for WV, suggesting the presence of a larger amount of additive genetic variation for these traits in this breeding population (Table 2). No difference was observed in the predictive abilities when using SNP sets including or not lower frequency SNPs. During cross-validation of genomic predictions a considerable variation was observed in the predictive abilities estimated across the different folds (Additional file 2: Table S3). This variation was larger for *E. benthamii*, where the predictive ability across folds ranged from a low −0.058 to 0.415 using BRR for DBH, with an average of 0.162 with a standard error (SE) of ±0.044. In *E. pellita*, the variation was smaller, with estimates ranging from 0.358 to 0.550 for DBH, with the ten-fold average equal to 0.441 ± 0.019 (Additional file 2: Table S3).

### Impact of variable numbers of SNPs on genomic predictions

**Fig. 2:**
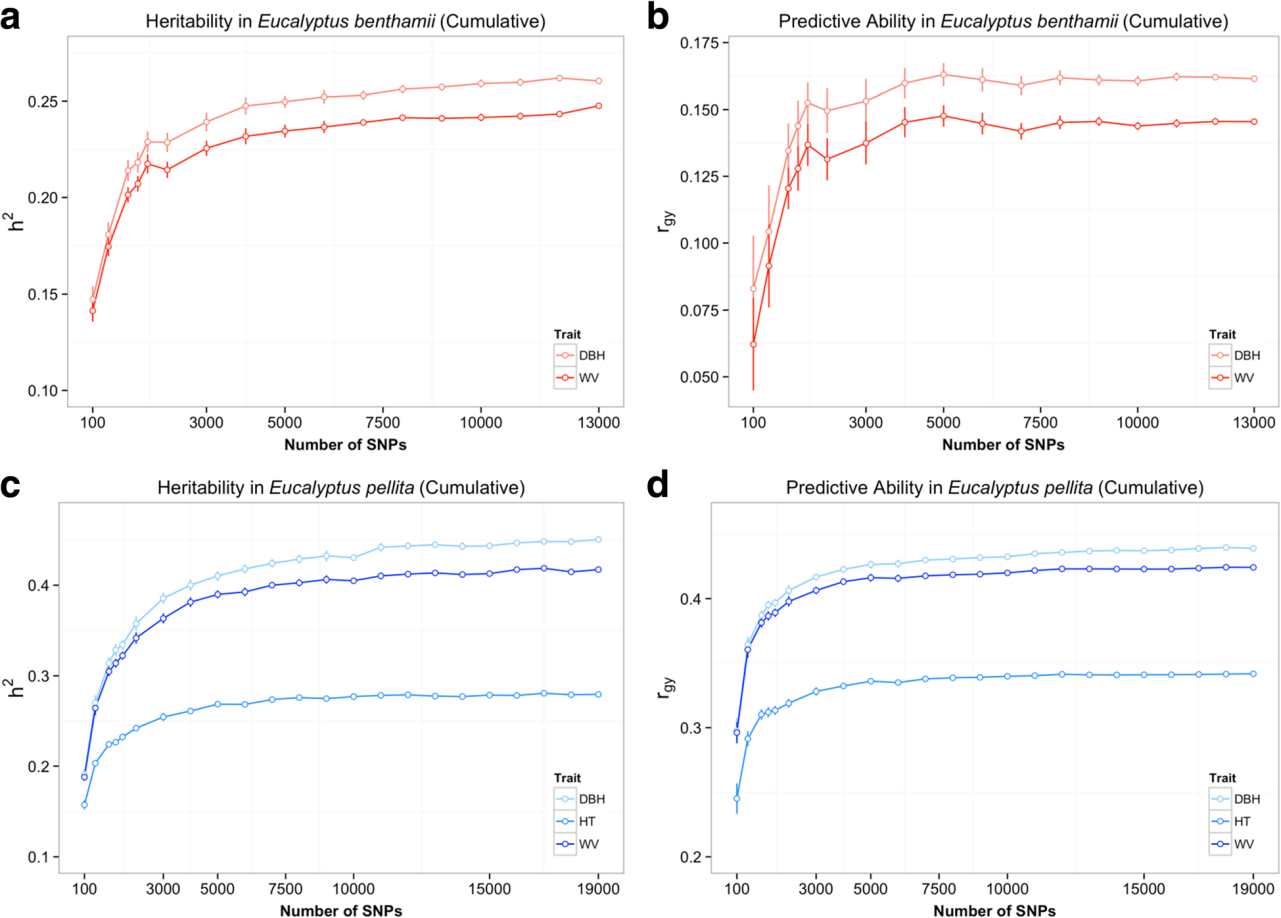
Estimates of heritability (*h* ^*2*^) and predictive ability (*r*_*gy*_) with increasing numbers of SNPs for different traits using a cumulative approach to SNP sampling. **a** and **b** estimates of *h* ^*2*^ and *r*_*gy*_ for *E. benthamii*, respectively; **c** and **d** estimates of *h* ^*2*^ and *r*_*gy*_ for *E. pellita*, respectively

Based on results of the different prediction methods, we chose to use only BRR to evaluate the impact of different SNPs sampling schemes on the predictive abilities. Subsets with progressively increasing randomly selected numbers of SNPs stratified by chromosome were used to estimate genomic predictions. Estimates of predictive ability and heritability increased rapidly with increasing number of SNPs up to ~3000 for all traits in both populations, (Table 3, Fig. 2). Predictive abilities plateaued at approximately 5000 SNPs although heritabilities and predictive abilities still increased by 5 to 10% after that. Additionally, when less than 5000 SNPs were used, a much larger variation in predictive ability was seen across the validation folds. These results indicate that at least in these populations, models with ~5000 to 10,000 SNPs will provide predictive abilities equivalent to those obtainable by using all the available SNPs. The non-cumulative sampling approach yielded essentially the same results with a plateau at ~5000 SNPs, but showed a more spiky pattern of increasing predictive ability as more SNPs were fitted into the model (Additional file 2: Figure S2).

**Table 3:** Genomic estimates of narrow-sense heritabilities (*h* ^*2*^) and predictive abilities (*r*_*gy*_) for the *E. benthamii* and *E. pellita* breeding populations using different SNP sampling methods

SNP sampling method	*E. benthamii*					*E. pellita*						
	Number of SNPs	DBH		WV		Number of SNPs	DBH		HT		WV	
		*h* ^*2*^ (SE)	*r*_*gy*_ (SE)	*h* ^*2*^ (SE)	*r*_*gy*_ (SE)		*h* ^*2*^ (SE)	*r*_*gy*_ (SE)	*h* ^*2*^ (SE)	*r*_*gy*_ (SE)	*h* ^*2*^ (SE)	*r*_*gy*_ (SE)
All SNPs	13,787	0.267 (0.008)	0.162 (0.044)	0.243 (0.008)	0.146 (0.039)	19,506	0.455 (0.005)	0.441 (0.019)	0.283 (0.008)	0.342 (0.042)	0.418 (0.005)	0.425 (0.028)
Randomly selected	5000	0.250 (0.003)	0.163 (0.004)	0.234 (0.003)	0.148 (0.004)	5000	0.410 (0.006)	0.427 (0.003)	0.269 (0.003)	0.336 (0.002)	0.390 (0.004)	0.416 (0.003)
Randomly selected	3000	0.239 (0.005)	0.153 (0.008)	0.226 (0.004)	0.137 (0.008)	3000	0.385 (0.006)	0.417 (0.003)	0.254 (0.005)	0.328 (0.003)	0.363 (0.006)	0.406 (0.003)
Randomly selected	1500	0.229 (0.005)	0.153 (0.008)	0.217 (0.005)	0.137 (0.008)	1500	0.334 (0.005)	0.397 (0.003)	0.232 (0.004)	0.313 (0.003)	0.322 (0.004)	0.389 (0.003)
Randomly selected	500	0.181 (0.006)	0.104 (0.017)	0.174 (0.005)	0.091 (0.015)	500	0.270 (0.008)	0.364 (0.006)	0.203 (0.003)	0.291 (0.006)	0.264 (0.008)	0.361 (0.006)
Evenly spaced 10 Kbp	10,837	0.264 (0.007)	0.159 (0.046)	0.235 (0.007)	0.141 (0.041)	13,946	0.452 (0.004)	0.436 (0.019)	0.272 (0.009)	0.340 (0.042)	0.415 (0.010)	0.421 (0.028)
Evenly spaced 50 Kbp	6867	0.253 (0.007)	0.153 (0.041)	0.242 (0.006)	0.135 (0.035)	7619	0.472 (0.008)	0.440 (0.021)	0.286 (0.008)	0.339 (0.043)	0.439 (0.008)	0.421 (0.031)
Evenly spaced 100 Kbp	4634	0.252 (0.004)	0.146 (0.044)	0.241 (0.006)	0.141 (0.036)	4846	0.460 (0.006)	0.442 (0.024)	0.287 (0.007)	0.339 (0.041)	0.452 (0.008)	0.432 (0.031)
Evenly spaced 250 Kbp	2281	0.261 (0.004)	0.166 (0.039)	0.258 (0.005)	0.160 (0.029)	2297	0.374 (0.007)	0.414 (0.026)	0.271 (0.005)	0.328 (0.042)	0.360 (0.004)	0.400 (0.030)
Evenly spaced 500 Kbp	1203	0.212 (0.006)	0.131 (0.053)	0.199 (0.004)	0.116 (0.050)	1204	0.326 (0.004)	0.388 (0.026)	0.226 (0.004)	0.306 (0.043)	0.307 (0.005)	0.378 (0.033)
Evenly spaced 1 Mbp	610	0.196 (0.002)	0.111 (0.031)	0.178 (0.003)	0.097 (0.022)	609	0.256 (0.004)	0.364 (0.027)	0.203 (0.004)	0.307 (0.041)	0.260 (0.004)	0.365 (0.029)
Genic regions	7254	0.251 (0.008)	0.163 (0.045)	0.240 (0.006)	0.148 (0.037)	11,212	0.421 (0.007)	0.433 (0.020)	0.269 (0.008)	0.340 (0.042)	0.394 (0.005)	0.426 (0.028)
Intergenic regions	6533	0.253 (0.008)	0.152 (0.046)	0.232 (0.005)	0.131 (0.046)	8294	0.449 (0.007)	0.432 (0.021)	0.289 (0.009)	0.340 (0.041)	0.414 (0.006)	0.410 (0.030)
SNPs in LE (LD-pruning)	10,460	0.274 (0.011)	0.174 (0.043)	0.256 (0.010)	0.161 (0.039)	10,984	0.425 (0.010)	0.426 (0.024)	0.275 (0.007)	0.339 (0.041)	0.404 (0.006)	0.413 (0.031)

*DBH, cm* (Diameter at Breast Height), *HT, m* (Total Height) and *WV, m* ^*3*^ (Wood Volume), *SE* (Standard Error)

### Impact of variable position-based SNP sampling methods

Overall, no difference was seen in the estimates of heritabilities and predictive abilities when different position-based SNP sampling schemes were used, as long as the total number of SNPs was close to 5000 (Table 3, Fig. 2). The predictive abilities estimated with a subset of evenly spaced SNPs every 1 Mbp windows (610 SNPs in *E. benthamii* and 609 SNPs in *E. pellita*), were slightly higher than those using 500 randomly sampled SNPs (Table 3). Although these results indicate that the number, and not the position of SNPs, determines the accuracy of predictions, they also suggest that even distribution might provide a small-added advantage when compared to random sampling. No significant differences in predictions were seen for any trait in both species when SNPs located in genic versus intergenic regions were used, and the predictions were equivalent to those obtained by random sampling of equivalent numbers of SNPs. The same result was observed with the LD-pruning approach, where estimates of predictive ability were similar either using LD-pruned SNPs in LE or all polymorphic SNPs (Table 3). There was no difference observed in the estimates of variance components when different sets of SNPs sampled based on position in the genome were used (Additional file 3).

When only SNPs located on single chromosomes were used, heritabilities dropped on average by 30–45% when compared to using all SNPs (e.g. for WV from 0.243 to 0.177 in *E. benthamii*; from 0.418 to 0.244 in *E. pellita*), indicating that genome-wide marker coverage is critical for capturing the additive genetic variance (Table 4). The predictive abilities using SNPs on single chromosomes were similar across chromosomes and also dropped on average by 15–30% when compared to using all SNPs (Table 4). However, when the heritabilities and predictive abilities provided by single chromosomes were compared to those obtained using equivalent numbers of randomly sampled SNPs subsets, no appreciable differences were seen. This result indicates that the drop in predictive ability is most likely due to the small number of SNPs per chromosome (average of 1253 for *E. benthamii* and 1773 for *E. pellita*) and not to the fact that they are located on a single chromosome. We did not have sufficient numbers of SNPs on a single chromosome to compare to the larger random subsets of 3000 or 5000 to see the effect on predictions.

**Table 4:** Genomic estimates of narrow-sense heritabilities (*h* ^*2*^) and predictive abilities (*r*_*gy*_) for the *E. benthamii* and *E. pellita* breeding populations using chromosome-specific SNP sets

	*E. benthamii*					*E. pellita*						
Chr	Number of SNPs	DBH		WV		Number of SNPs	DBH		HT		WV	
		*h* ^*2*^ (SE)	*r*_*gy*_ (SE)	*h* ^*2*^ (SE)	*r*_*gy*_ (SE)		*h* ^*2*^ (SE)	*r*_*gy*_ (SE)	*h* ^*2*^ (SE)	*r*_*gy*_ (SE)	*h* ^*2*^ (SE)	*r*_*gy*_ (SE)
1	848	0.162 (0.004)	0.070 (0.048)	0.161 (0.003)	0.071 (0.037)	1329	0.240 (0.004)	0.336 (0.034)	0.223 (0.006)	0.327 (0.042)	0.241 (0.004)	0.337 (0.031)
2	1672	0.186 (0.003)	0.085 (0.036)	0.183 (0.004)	0.071 (0.034)	2245	0.228 (0.004)	0.313 (0.033)	0.188 (0.004)	0.272 (0.040)	0.218 (0.004)	0.303 (0.036)
3	1544	0.195 (0.004)	0.170 (0.036)	0.207 (0.004)	0.185 (0.040)	2026	0.282 (0.007)	0.363 (0.042)	0.172 (0.003)	0.282 (0.046)	0.267 (0.006)	0.355 (0.043)
4	886	0.180 (0.004)	0.134 (0.036)	0.171 (0.004)	0.104 (0.027)	1303	0.256 (0.008)	0.315 (0.045)	0.203 (0.003)	0.271 (0.044)	0.251 (0.009)	0.294 (0.049)
5	1356	0.195 (0.004)	0.123 (0.051)	0.190 (0.004)	0.123 (0.052)	1872	0.303 (0.006)	0.379 (0.037)	0.227 (0.007)	0.325 (0.044)	0.277 (0.006)	0.353 (0.040)
6	1440	0.166 (0.004)	0.090 (0.040)	0.157 (0.002)	0.063 (0.033)	2012	0.277 (0.007)	0.375 (0.031)	0.197 (0.004)	0.294 (0.040)	0.274 (0.008)	0.369 (0.037)
7	1207	0.219 (0.006)	0.187 (0.051)	0.210 (0.006)	0.158 (0.046)	1594	0.226 (0.003)	0.337 (0.031)	0.168 (0.003)	0.241 (0.047)	0.217 (0.003)	0.323 (0.038)
8	1771	0.183 (0.005)	0.082 (0.063)	0.168 (0.004)	0.059 (0.050)	2583	0.212 (0.006)	0.306 (0.038)	0.185 (0.003)	0.267 (0.040)	0.222 (0.004)	0.316 (0.037)
9	940	0.170 (0.003)	0.121 (0.035)	0.164 (0.004)	0.100 (0.033)	1381	0.228 (0.004)	0.330 (0.020)	0.182 (0.004)	0.285 (0.034)	0.233 (0.006)	0.332 (0.027)
10	1034	0.152 (0.002)	0.059 (0.037)	0.150 (0.002)	0.047 (0.041)	1448	0.218 (0.003)	0.339 (0.037)	0.184 (0.004)	0.292 (0.050)	0.224 (0.004)	0.353 (0.041)
11	1089	0.195 (0.004)	0.138 (0.040)	0.193 (0.006)	0.143 (0.041)	1713	0.250 (0.005)	0.338 (0.024)	0.201 (0.005)	0.304 (0.042)	0.258 (0.006)	0.350 (0.026)
All	13,787	0.267 (0.008)	0.162 (0.044)	0.243 (0.008)	0.146 (0.039)	19,506	0.455 (0.005)	0.441 (0.019)	0.283 (0.008)	0.342 (0.042)	0.418 (0.005)	0.425 (0.028)

*DBH, cm* (Diameter at Breast Height), *HT, m* (Total Height) and *WV, m* ^*3*^ (Wood Volume), *SE* (Standard Error)

### Impact of relatedness between training and validation sets

**Fig. 3:**
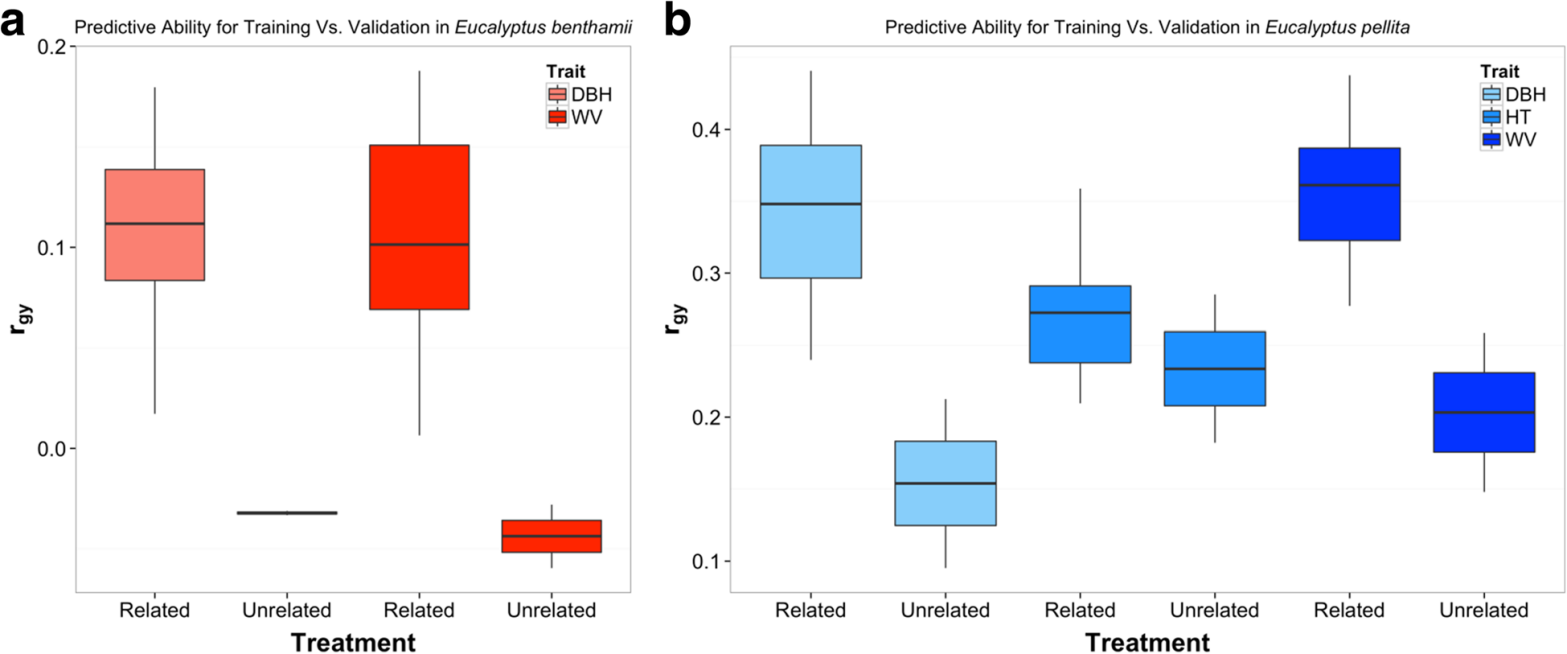
Estimates of predictive ability (*r*_*gy*_) with different levels of relatedness between training and validation sets. **Related:** random allocation of individuals to training and validation sets**; Unrelated:** individuals were split into training and validation sets by minimizing relatedness between sets based on a principal component analysis (**a**) *E. benthamii* and (**b**) *E. pellita*

To assess the relative contribution of relatedness to the predictive ability (as opposed to short-range historical LD between SNPs and QTL), GS models were fitted trying to minimize relatedness between training and validation sets based on genetic differentiation determined by a PCA (Additional file 2: Figure S3). Predictive ability obtained when minimizing relatedness was null for *E. benthamii* (Fig. 3a) (e.g. from 0.108 to −0.032 for DBH) and reduced approximately by half for *E. pellita* (e.g. from 0.348 to 0.154 for DBH) compared to the predictive abilities achieved when the same number of individuals were used to build the model without controlling for relatedness (Fig. 3b). These results suggest that predictions in the *E. benthamii* population were fully dependent on relatedness, while in *E. pellita* some short-range SNP-QTL LD might be contributing to predictions, although relatedness also seems to be the main driver.

### Association genetics models comparison

**Fig. 4:**
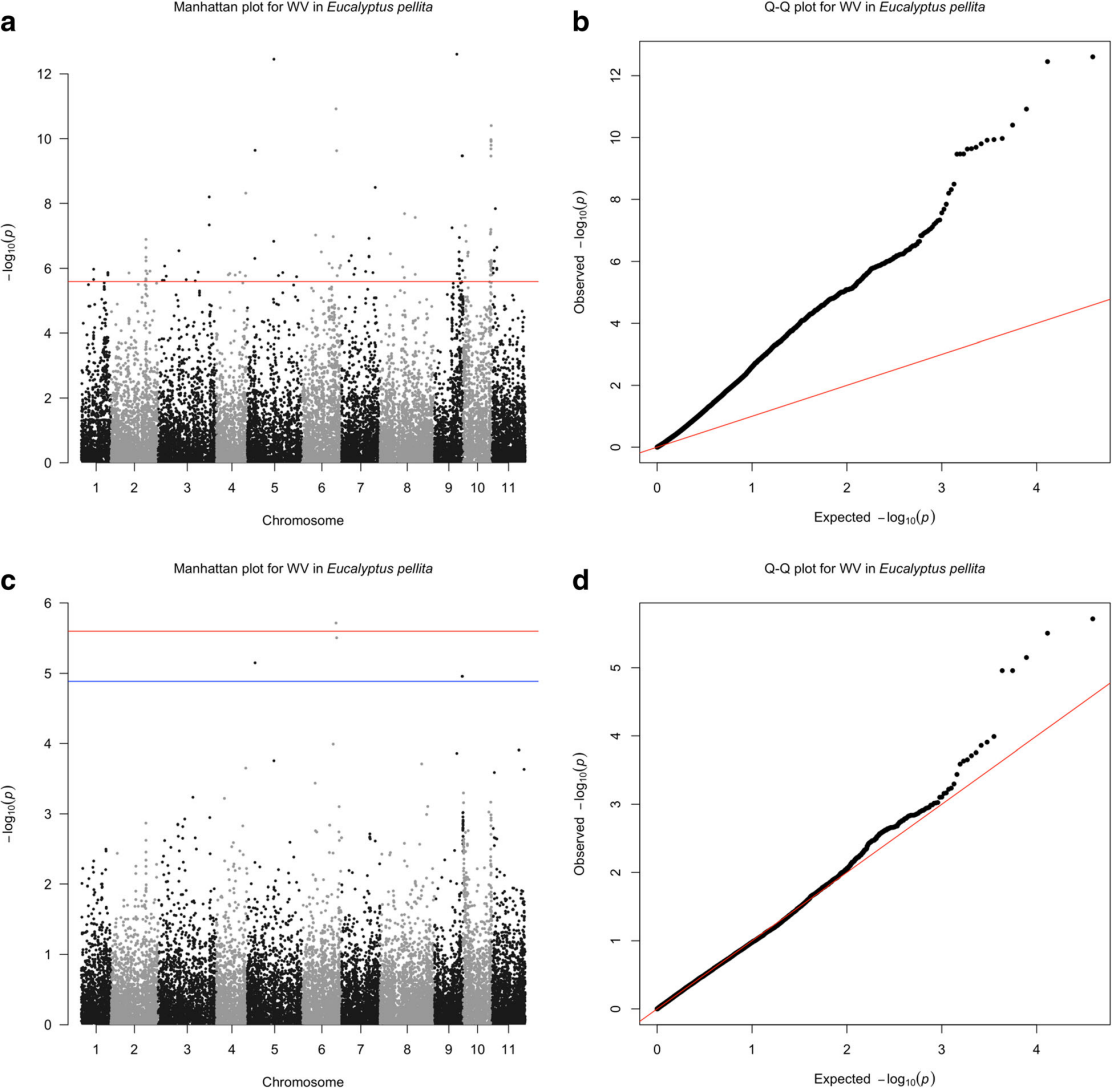
Manhattan and Quantile-quantile (Q-Q) plots for wood volume (WV) in *E. pellita*. **a** and **b** represent the Manhattan and the Q-Q plots, respectively, for LMA model adjusted for block and population structure covariates. **c** and **d** represent the Manhattan and Q-Q plots, respectively, for the MLMA model adjusted for block and population structure covariates, and for the genomic relationship matrix. *Red line* indicates Bonferroni-corrected threshold with an experimental type I error rate at α = 0.05 and *blue line* indicates false discovery rate (FDR) at 5%

GWAS under an LMA model, i.e. without the introduction of a GRM, resulted in a large number of associations, most or all of them likely spurious. For example, with only block as a covariate in the model, the number of SNPs associated with wood volume (WV) in *E. pellita* was 249. When the population structure was included as covariate, the number of associated SNPs was reduced to 120 (Fig. 4a, red line). The quantile-quantile (Q-Q) plot exhibited in Fig. 4b shows the inappropriateness of the LMA model without the GRM, as the observed and expected *P*-values differed considerably for a large number of SNPs. When the genomic relationship matrix, block and structure effects were included in the MLMA model, five significant associations (Fig. 4c, blue line) were detected using a FDR of 0.05 (Additional file 2: Table S4). All these five significant SNPs have low allele frequency (MAF < 0.005). Nevertheless, when a more stringent adjustment for multiple testing was used (Bonferroni 5%), only one significant association persisted for volume in *E. pellita* (Fig. 4c, red line). In the MLMA model adjusted for the GRM, population structure and block covariates, most *P*-values were consistent with the expected ones along the diagonal in the Q-Q plot, indicating suitability of this GWAS model (Fig. 4d). Furthermore, the model built with GRM reduced considerably the number of significant associations, likely removing spurious associations. The single SNP associated with volume in *E. pellita* on chromosome 6 (Fig. 4c, red line) is located in an exonic region of a gene whose function is involved in a plant-type cell wall cellulose biosynthetic process (Additional file 2: Table S4). In *E. benthamii*, no significant associations were found when the GRM was included in the model.

## Discussion

This study makes a further step towards the experimental assessment of whole-genomic prediction of complex traits in species of forest trees in general and of *Eucalyptus* in particular. Our results corroborate previous reports in forest trees showing encouraging perspectives of using genome-wide SNP data to capture large proportions of trait heritability and predict traits such as height and diameter growth with accuracies as good as or better than those attainable by conventional phenotypic selection.

### Genomic heritabilities and predictions

Genomic heritabilities, irrespective of the method used, were generally lower than the pedigree-based estimates, with the exception of HT in *E. benthamii* (Table 2). Genomic heritability is considered to better reflect the true genetic relationships among individuals and as such, it corresponds to the proportion of phenotypic variance that can be explained by regression on molecular markers. The genomic heritability and trait heritability are expected to be equal only when all causal variants are typed. Additionally, when close relatives sharing long chromosome segments are analyzed, high prediction accuracy and very small bias in genomic heritability estimates are expected [47]. Given the relatively long-range LD and relatedness in our populations, our estimates of genomic heritability should closely reflect the amount of additive genetic variance for the traits measured. Genomic heritabilities lower than the pedigree-based estimates were also reported in open-pollinated families of spruce [19, 21]. Pedigree-based heritability estimates from open-pollinated families could be inflated due to the presence of full-sibs or selfs and the inability of these estimates to disentangle the non-additive from the additive genetic components [48]. For *E. pellita,* pedigree-based heritability could not be estimated. However, by using the SNP data, heritability estimates were obtained that breeders would not otherwise have had access to.

Predictive abilities of growth traits using GBLUP and different Bayesian methods reached similar results for all traits, in line with previous reports in forest trees [16, 20, 22]. These results provide further evidence that growth traits in *Eucalyptus,* and likely for all forest trees, are complex in architecture, controlled by a large number of small effect loci and fit adequately the infinitesimal model. The predictive ability estimates obtained for growth traits in *E. pellita* (0.34–0.44) using GBLUP were slightly lower than those reported for *E. grandis x E. urophylla* (0.46–0.55) [14]. For *E. benthamii*, predictive abilities were lower (~0.16), possibly the result of (i) the larger effective population size; (ii) the relatively limited number of individuals used for model training (only ~500); and (iii) the limited genetic diversity available in this species and particularly so in this introduced population in Brazil, also indicated by the low heritability found in our study as well as in others with similar germplasm [6]. From the applied breeding standpoint however, the genomic predictive abilities were as good as or better than the predictive abilities based on phenotypic data.

Prediction models using ~5000 SNPs provided predictive abilities almost equivalent to using all available SNPs for all traits and no difference was observed using different sets of SNPs. These results suggest that genomic prediction is largely driven by relatedness such that once a certain number of randomly sampled SNPs across the genome are used, suitable predictive ability is reached. This outcome indicates that low-density SNP chips could be contemplated as a way to reduce cost of GS in line to what has been the case for domestic animals [26, 49]. It is expected, however, that genomic predictions will decay over generations due to the combined effect of recombination and selection on the patterns of LD [50], unless continuous model retraining strategies are adopted [12]. At this point, therefore, it is not clear whether the use of smaller SNP subsets is warranted for the long-term implementation of GS in *Eucalyptus*. A better assessment will be possible when predictions are carried out across breeding generations testing variable SNP densities.

We observed a major impact of relatedness on predictions, more so in *E. benthamii* than *E. pellita* (Fig. 3) consistent with theoretical expectations [24] and previous experimental results in forest trees [14, 18, 19]. The relative contributions of historical LD and relatedness are however difficult to disentangle. Predictive ability can be high even in the absence of LD when markers capture genetic relationships, but it will be even greater if markers are in LD with causal loci [24]. The extent of LD detected in these populations reflected their differences in evolutionary and breeding history. A faster genome-wide LD decay was observed in *E. benthamii* (7.7 Kb, Fig. 1a) than in *E. pellita* (25.6 Kbp, Fig. 1c). While the *E. benthamii* population is derived from seeds collected in wild stands and its LD was similar to that found in natural populations of *E. grandis* (≈4–6 Kb) [51], the *E. pellita* population comes from a clonal seed orchard established with advanced selections such that a smaller effective population size and more extensive LD was expected.

The presence of some level of short-range historical LD could in part explain why predictions were still reasonable in *E. pellita* even after attempting to minimize relatedness between training and validation sets (Fig. 3b). However, another possibility is that our attempt to decrease relatedness was not completely efficient. To evaluate these alternative hypotheses we compared the predictive abilities obtained using the same number of markers concentrated on a single chromosome (capturing largely the effect of relatedness), versus distributed genome-wide (capturing relatedness and LD). Assuming an infinitesimal model in which growth traits are controlled by many QTLs with small effects distributed genome-wide, the difference between these two sets could be tentatively taken as the contribution of historical LD to predictions. An increase of 22 to 35% in predictive ability was seen (e.g. 0.306 versus 0.414 for DBH) when genome-wide SNPs were used, suggesting that some short-range historical LD between markers and causal loci could be accounted for in this population. Overall, our results corroborate previous reports on the major impact of relatedness on genomic prediction and further highlight the importance of properly planning the populations on which GS models will be trained and those where the models will be applied. If the training population is more or less related to the validation population than the future selection candidates, then the expected outcome of implementing genomic selection will be over- or underestimated, respectively.

### GWAS versus genomic prediction in breeding populations

The objective of our GWAS was to assess the value of this approach in closed breeding populations under selection and compare it to whole-genome prediction from the standpoint of how much genetic variation could be captured for practical breeding. After duly controlling for population structure and experimental fixed effects, and applying experiment-wide corrections for multiple tests, we identified only one significant association for volume growth in *E. pellita* (Fig. 4c). Despite the relatively larger population size (*n* = 732) when compared to populations used in previous GWAS in forest trees (typically between ~300 and ~700 individuals), our results are consistent with the fact that very few associations were also found for growth in all those reported GWAS to date [52–59]. Population sizes used have been small, such that experiments have suffered from low power to detect the likely large number of small effect loci controlling growth. Integrating linkage mapping data from bi-parental pedigrees with association populations has been attempted but results have not improved and only a handful of associations have been found, again explaining very little of the genetic variation [56, 57, 59]. Our direct comparison between GS and GWAS is novel and more explicitly corroborates the fact that while genome-wide regression is able to account for large proportions of the pedigree-heritability (e.g. 73% for DBH in *E. benthamii*) and provide useful phenotype predictions, very little of the heritability is captured into significant associations using the GWAS approach. Reasons for this major discrepancy are not surprising and have been widely discussed in the plant, animal and human literature [60–62]. They derive essentially from the fact that GWAS by principle, relies on the application of stringent significance tests to declare an association. These very stringent tests typically result in only the largest effect QTLs being found, while the vast majority have too small an effect to be detectable in the limited power GWAS populations used. If no major effect exists, then no associations are found, which is most likely the case of the limited association results for growth targeted in our study.

A potential criticism to our GWAS is the fact that it was carried out in a breeding population with limited diversity and not in a canonical GWAS population sampled from the wild. GWAS studies for growth traits in forest trees have in fact targeted collections of trees derived from natural populations sampling large amounts of diversity. The goal of those studies has been to detect associations that would potentially allow gene discovery or even the identification of the elusive QTN (quantitative trait nucleotide) [63]. However, notwithstanding the fact that very few associations were found for growth traits in those GWAS, explaining overall negligible fractions of trait heritability, it is not clear yet how marker-trait associations detected in undomesticated tree populations, genetically distant from improved germplasm, would be converted into useful information to breeding practice. This, in fact, has not been demonstrated yet in forest trees. Targeted alleles found by GWAS in natural populations might contribute relatively negligible effects, be already fixed or simply not be sampled in existing breeding populations [64]. On the other hand, although genetic variation available in breeding populations is in principle more limited, associations detected in genetically improved material should be more relevant to breeding. A recent GWAS in a *Eucalyptus* breeding population reported promising results using a regional heritability mapping, an approach able to capture both common and rare allelic effects that individually contribute too little variance to be detected by conventional GWAS [58]. The availability of GWAS data could be valuable to improve genomic predictions accuracies by assigning locus- or trait-specific priors to genomic prediction models [65], as recently shown in rice [66].

## Conclusions

This study contributes further experimental data supporting the positive prospects of genomic selection to predict complex traits such as height and diameter growth in forest trees with accuracies equivalent or superior to those achievable by phenotypic selection. We show that genetic relatedness captured by the SNP data between training and validation populations and, by extension, to future selection candidates, is what will most likely determine the successful use of genomic selection in *Eucalyptus* breeding. We also conclude that more important to GS than the number and position of the SNPs fitted in the model, is the extensive LD created in closed breeding populations with small effective population sizes. Lower density SNP panels with ~5000 to 10,000 SNPs, distributed across the genome, should provide a good compromise between genotyping costs and predictive ability in such standard breeding populations advanced by open pollinated breeding. However, further experiments are necessary to evaluate the performance of such SNP densities across generations of breeding. Our results also illustrate the superiority of the whole-genome regression approach in accounting for large proportions of the heritability in contrast to the limited value of the local GWAS approach for breeding applications. To provide useful GWAS data toward breeding for growth traits in *Eucalyptus* and likely in all forest trees, it will be necessary first to massively increase the sample size, such that sufficient power is reached to detect at least part of the slightly larger effects segregating in the target breeding population. In the meantime, the encouraging results of genomic prediction that we, and others, have shown in this and other studies should probably receive greater attention if the objective is to impact breeding practice.

## Additional files

Additional file 1: Description of the Bayesian methods used for genomic predictions. (DOCX 25 kb)

Additional file 2: Supplementary Tables S1 through S4 and supplementary Figures S1 through S4. Supplementary figure legends are contained within the file. (DOCX 1558 kb)

Additional file 3: Estimates of additive genetic variance (σ^2^a) and residual variance (σ^2^e) obtained with different prediction methods, different position-based SNP sampling methods and sampling related or unrelated individuals in the *E. benthamii* and *E. pellita* breeding populations. (XLS 55 kb)
