# Supplementary material for: Genomic prediction in contrast to a genome-wide association study in explaining heritable variation of complex growth traits in breeding populations of Eucalyptus
Source: BMC Genomics. 2017 Jul 11;18:524. doi: 10.1186/s12864-017-3920-2 (PMC5504793; doi:10.1186/s12864-017-3920-2)
Supplement: Supplementary file 1 — Description of the Bayesian methods used for genomic predictions. (DOCX 25 kb) [file 12864_2017_3920_MOESM1_ESM.docx]

**Additional file 1**. Description of the Bayesian methods used for genomic predictions.

The *Bayesian Ridge-Regression* (BRR) is the Bayesian version of GBLUP [1] and assumes that all marker effects have the same variance component. Consequently, markers with the same allele frequency contribute equally to the genetic variance. For the BRR model it was assumed that:

$$m_{i}|\sigma_{m}^{2}\sim N(0,\sigma_{m}^{2})$$

$$\sigma_{m}^{2}|S_{m},\nu_{m}\sim\chi^{-2}(S_{m},\nu_{m})$$

The *Bayes A* method was proposed by Meuwissen et al. [1] and later modified by Pérez and de los Campos [2] to reduce the influence of the hyperparameters and achieves better Bayesian learning. The Bayes A model assumes that marker effects have heterogeneous variances. In the Bayes A model it was assumed that:

$$m_{i}|\sigma_{m_{i}}^{2}\sim N(0,\sigma_{m_{i}}^{2})$$

$$\sigma_{m_{i}}^{2}|S_{m},\nu_{m}\sim\chi^{-2}(S_{m},\nu_{m})$$

$$S_{m}|r,s\sim G(r,s)$$

The *Bayes B* method was also proposed by Meuwissen et al. [1] and modified in Pérez and de los Campos [2] to achieve better Bayesian learning and to estimate the proportion of markers with null effect. This model is similar to Bayes A, and assumes that the markers have heterogeneous variance component. Additionally, it considers that a proportion of markers have non-null effects. This is in contrast to Bayes A, because the approach includes the selection of covariates (SNPs markers) that do not contribute to genetic variance. In the Bayes B model it was assumed that:

| $m_{i}\vert\sigma_{m_{i}}^{2}$ | $\sim N(0,\sigma_{m_{i}}^{2})$ | with probability equal 1**-**π |
| --- | --- | --- |
|  | =0 | with probability equal π |

$$\sigma_{m_{i}}^{2}|S_{m},\nu_{m}\sim\chi^{-2}(S_{m},\nu_{m})$$

$$S_{m}|r,s\sim G(r,s)$$

$$\pi\sim Beta(p_{0},\pi_{0})$$

The *Bayes Cπ* method proposed by Habier et al. [3] is derived from the Bayes C method and is similar to BRR. In this approach it is assumed that the marker effects have a common variance. However, Bayes Cπ includes marker selection with parameter π, which is defined as the probability of a SNP marker having a null effect. For the Bayes Cπ method it is assumed that:

| $m_{i}\vert\sigma_{m}^{2}$ | $\sim N(0,\sigma_{m}^{2})$ | with probability equal 1**-**π |
| --- | --- | --- |
|  | =0 | with probability equal π |

$$\sigma_{m}^{2}|S_{m},\nu_{m}\sim\chi^{-2}(S_{m},\nu_{m})$$

$$\pi\sim Beta(p_{0},\pi_{0})$$

The *Bayesian Lasso* (BL) method was proposed by Park and Casella [4] and was adapted for genomic prediction by de los Campos et al. [5]. Similar to Bayes A and Bayes B, the BL method assumes covariates with heterogeneous variance. The BL method does indirect marker selection, since the marginal distribution of the markers follows a double exponential distribution, providing strong shrinkage of the marker effects to close to zero for large number of markers. In the BL method it is assumed that:

$$m_{i}|\sigma_{e}^{2},\tau_{i}^{2}\sim N(0,\sigma_{e}^{2}\times\tau_{i}^{2})$$

$$\tau_{i}^{2}|\lambda\sim Exp(0.5\lambda^{2})$$

$$\lambda|r,s\sim G(r,s)$$

**References**

1. Meuwissen THE, Hayes BJ, Goddard ME. Prediction of total genetic value using genome-wide dense marker maps. Genetics. 2001;157:1819–29.

2. Pérez P, de los Campos G. Genome-wide regression and prediction with the BGLR statistical package. Genetics. 2014;198:483–95.

3. Habier D, Fernando RL, Kizilkaya K, Garrick DJ. Extension of the bayesian alphabet for genomic selection. BMC Bioinformatics. BioMed Central Ltd; 2011;12:186.

4. Park T, Casella G. The Bayesian Lasso. J. Am. Stat. Assoc. 2008;103:681–6.

5. de los Campos G, Naya H, Gianola D, Crossa J, Legarra A, Manfredi E, et al. Predicting quantitative traits with regression models for dense molecular markers and pedigree. Genetics. 2009;182:375–85.
