# Supplementary material for: Genomic prediction in contrast to a genome-wide association study in explaining heritable variation of complex growth traits in breeding populations of Eucalyptus
Source: BMC Genomics. 2017 Jul 11;18:524. doi: 10.1186/s12864-017-3920-2 (PMC5504793; doi:10.1186/s12864-017-3920-2)
Supplement: Supplementary file 2 — Supplementary Tables S1 through S4 and supplementary Figures S1 through S4. Supplementary figure legends are contained within the file. (DOCX 1558 kb) [file 12864_2017_3920_MOESM2_ESM.docx]

**Additional file 2**

**Table S1:** Numbers of SNPs and average distances between SNPs for the variable window sizes used to select evenly spaced SNP subsets for genomic prediction in *E. benthamii* and *E. pellita*.

**Table S2:** Linkage Disequilibrium (LD) estimates and genome-wide pattern of decay of LD up to pairwise SNP distance of 100 Kbp including rare alleles (MAF > 0) or not (MAF ≥ 5%) for the *E. benthamii* and *E. pellita* populations.

**Table S3:** Predictive ability of growth traits of different 10-fold cross-validation using Bayesian Ridge-Regression (BRR) models in *E. benthamii* and *E. pellita* populations.

**Table S4:** Significant SNP associations with wood volume trait in *E. pellita* using MLMA model adjusted for block, population structure covariates and genomic relationship matrix.

**Figure S1: Distribution of the numbers of SNPs for variable filtering criteria and MAF classes.** (A) Distribution of the number of SNPs retained in variable filtering criteria, and (B) Distribution of the number of SNPs into MAF classes for *E. benthamii* and *E. pellita* for CR ≥ 90% and MAF > 0 (CR, Call Rate; MAF, Minimum Allele Frequency; LE, Linkage Equilibrium).

**Figure S2: Estimates of heritability (*h^2^*) and predictive ability (*r_gy_*) with increasing numbers of SNPs for different traits using a non-cumulative approach to SNP sampling.** (A) and (B) estimates of *h^2^* and *r_gy_* for *E. benthamii*, respectively; (C) and (D) estimates of *h^2^* and *r_gy_* for *E. pellita*, respectively.

**Figure S3: Principal component analysis (PCA) of the 484 trees of *E. benthamii* (A) and 706 trees of *E. pellita* (B) used to split training and validation sets.** For *E. benthamii* 310 (red) and 174 (pink) individuals were used as training and validation sets. In *E. pellita*, the number of individuals used in each set were 192 (dark blue) and 514 (light blue).

**Table S1:** Numbers of SNPs and average distances between SNPs for the variable window sizes used to select evenly spaced SNP subsets for *E. benthamii* and *E. pellita*.

| **Selected window (Kbp)** |  | ***E. benthamii*** | |  | ***E. pellita*** | |
| --- | --- | --- | --- | --- | --- | --- |
|  |  | **Average distance between markers (Kbp)** | **Number of SNPs** |  | **Average distance between markers (Kbp)** | **Number of SNPs** |
| 10 |  | 55.7 | 10,837 |  | 43.2 | 13,946 |
| 50 |  | 87.4 | 6,867 |  | 78.6 | 7,619 |
| 100 |  | 130 | 4,634 |  | 124 | 4,846 |
| 250 |  | 265 | 2,281 |  | 263 | 2,297 |
| 500 |  | 504 | 1,203 |  | 503 | 1,204 |
| 1,000 |  | 1,000 | 610 |  | 1,002 | 609 |

**Table S2:** Linkage Disequilibrium (LD) estimates and genome-wide pattern of decay of LD up to pairwise SNP distance of 100 Kbp including rare alleles (MAF > 0) or not (MAF ≥ 5%) for the *E. benthamii* and *E. pellita* populations.

| **LD measurement** | ***E. benthamii*** | |  | ***E. pellita*** | |
| --- | --- | --- | --- | --- | --- |
|  | **MAF > 0** | **MAF ≥ 5%** |  | **MAF > 0** | **MAF ≥ 5%** |
| Number of SNPs | 13,787 | 7,563 |  | 19,506 | 12,483 |
| Number of SNPs pairwise | 9,157,068 | 2,817,210 |  | 18,146,366 | 7,494,379 |
| Mean *r^2^* all data | 0.0169 | 0.0164 |  | 0.0167 | 0.0187 |
| Mean *r^2^S* all data | 0.0161 | 0.0149 |  | 0.0158 | 0.0176 |
| Mean *r^2^V* all data | 0.0117 | 0.0063 |  | 0.0067 | 0.0063 |
| Mean *r^2^VS* all data | 0.0117 | 0.0063 |  | 0.0067 | 0.0063 |
| Mean *r^2^* in 100 Kbp | 0.1413 | 0.2284 |  | 0.2713 | 0.3173 |
| Mean *r^2^S* in 100 Kbp | 0.1372 | 0.2225 |  | 0.2654 | 0.3123 |
| Mean *r^2^V* in 100 Kbp | 0.0966 | 0.1443 |  | 0.1790 | 0.2142 |
| Mean *r^2^VS* in 100 Kbp | 0.0966 | 0.1444 |  | 0.1788 | 0.2142 |
| Mean *r^2^* in 50 Kbp | 0.2015 | 0.2955 |  | 0.3331 | 0.3697 |
| Mean *r^2^S* in 50 Kbp | 0.1966 | 0.2897 |  | 0.3280 | 0.3659 |
| Mean *r^2^V* in 50 Kbp | 0.1451 | 0.2050 |  | 0.2437 | 0.2795 |
| Mean *r^2^VS* in 50 Kbp | 0.1451 | 0.2051 |  | 0.2435 | 0.2794 |
| *r^2^* <0.2 within (Kbp) | 15.622 | 40.693 |  | 70.595 | 112.678 |
| *r^2^S* <0.2 within (Kbp) | 14.755 | 38.096 |  | 66.237 | 106.198 |
| *r^2^V* <0.2 within (Kbp) | 7.708 | 14.515 |  | 25.614 | 35.888 |
| *r^2^VS* <0.2 within (Kbp) | 7.708 | 14.526 |  | 25.556 | 35.862 |
| Half-decay distance (Kbp) *r^2^* | 12.217 | 31.729 |  | 55.106 | 87.923 |
| Half-decay distance (Kbp) *r^2^S* | 11.543 | 29.701 |  | 51.706 | 82.865 |
| Half-decay distance (Kbp) *r^2^V* | 6.059 | 11.348 |  | 20.033 | 28.042 |
| Half-decay distance (Kbp) *r^2^VS* | 6.059 | 11.365 |  | 19.987 | 28.022 |
| Average of SNPs by Chr. | 1,253.4 | 687.5 |  | 1,773.3 | 1,134.8 |

LD estimates with classical measure of the squared correlation of allele frequencies at diallelic loci (*r^2^*), adjusted for population structure (*r^2^S*) and relatedness (*r^2^V*), and adjusted for both (*r^2^VS*).

**Table S3:** Predictive ability of growth traits of different 10-fold cross-validation using Bayesian Ridge-Regression (BRR) models in *E. benthamii* and *E. pellita* populations.

| **BRR *r_gy_*** | ***E. benthamii*** | |  | ***E. pellita*** | | |
| --- | --- | --- | --- | --- | --- | --- |
| **Fold** | **DBH** | **WV** |  | **DBH** | **HT** | **WV** |
| fold1 | 0.044 | -0.015 |  | 0.482 | 0.356 | 0.506 |
| fold2 | 0.251 | 0.294 |  | 0.431 | 0.448 | 0.444 |
| fold3 | 0.415 | 0.335 |  | 0.550 | 0.540 | 0.536 |
| fold4 | 0.180 | 0.195 |  | 0.358 | 0.236 | 0.309 |
| fold5 | 0.205 | 0.142 |  | 0.416 | 0.421 | 0.395 |
| fold6 | 0.199 | 0.196 |  | 0.502 | 0.355 | 0.493 |
| fold7 | 0.012 | 0.046 |  | 0.373 | 0.070 | 0.263 |
| fold8 | 0.268 | 0.156 |  | 0.397 | 0.302 | 0.382 |
| fold9 | 0.104 | 0.164 |  | 0.459 | 0.427 | 0.432 |
| fold10 | -0.058 | -0.056 |  | 0.439 | 0.261 | 0.488 |
| Mean | 0.162 | 0.146 |  | 0.441 | 0.342 | 0.425 |
| SE | 0.044 | 0.039 |  | 0.019 | 0.042 | 0.028 |

**Table S4:** Significant SNP associations with wood volume trait in *E. pellita* using MLMA model adjusted for block, population structure covariates and genomic relationship matrix.

| **SNP^*^** | **MAF** | ***P*-value** | **Bonferroni^a^** | **FDR^b^** | **Annotation^c^** | **Description^c^** | **Function^d^** |
| --- | --- | --- | --- | --- | --- | --- | --- |
| EuBR06s46273728 | 0.00410 | 1.91E-06 | 0.0373 | 0.0303 | Eucgr.F03806.1 / AT1G77460.1 | Armadillo/beta-catenin-like repeat; C2 calcium/lipid-binding domain (CaLB) protein. | Plant-type cell wall cellulose biosynthetic process and unidimensional cell growth. |
| EuBR06s47094282 | 0.00274 | 3.11E-06 | 0.0606 | 0.0303 | Orysa\|LOC_Os06g43560.1_GX6P | Phox domain-containing protein, putative, expressed LOC_Os06g43560.1. | Phosphatidylinositol binding. |
| EuBR05s10629849 | 0.00478 | 7.07E-06 | 0.1379 | 0.0430 | Eucgr.E01008.1 / AT1G11050.1 | Non-specific serine/threonine protein kinase / Threonine-specific protein kinase or Protein kinase superfamily protein. | Catalytic activity (ATP + a protein = ADP + a phosphoprotein). |
| EuBR09s37575166 | 0.00342 | 1.10E-05 | 0.2149 | 0.0430 | Eucgr.I02627.1 / AT1G32750.1 | Transcription initiation factor TFIID \| HAC13 protein (HAC13). | Chromatin modification, DNA mediated transformation, regulation of transcription, DNA-templated. |
| EuBR09s38076481 | 0.00342 | 1.10E-05 | 0.2149 | 0.0430 | Eucgr.I02694.1 / AT3G19720.1 | Dynamin-like protein ARC5 \| P-loop containing nucleoside triphosphate hydrolases superfamily protein. | Catalytic activity (GTP + H2O = GDP + phosphate). |

**^*^**SNP name: e.g. EuBR06s46273728, SNP on chromosome 6 at position 46,273,728 bp

^a^Bonferroni-corrected threshold with an experimental type I error rate at α = 0.05.

^b^False Discovery Rate (FDR) threshold at 5%.

^c^Annotation information based on BLASTx in Phytozome for the *Eucalyptus grandis* genome

^d^Function information based on UniProt database.


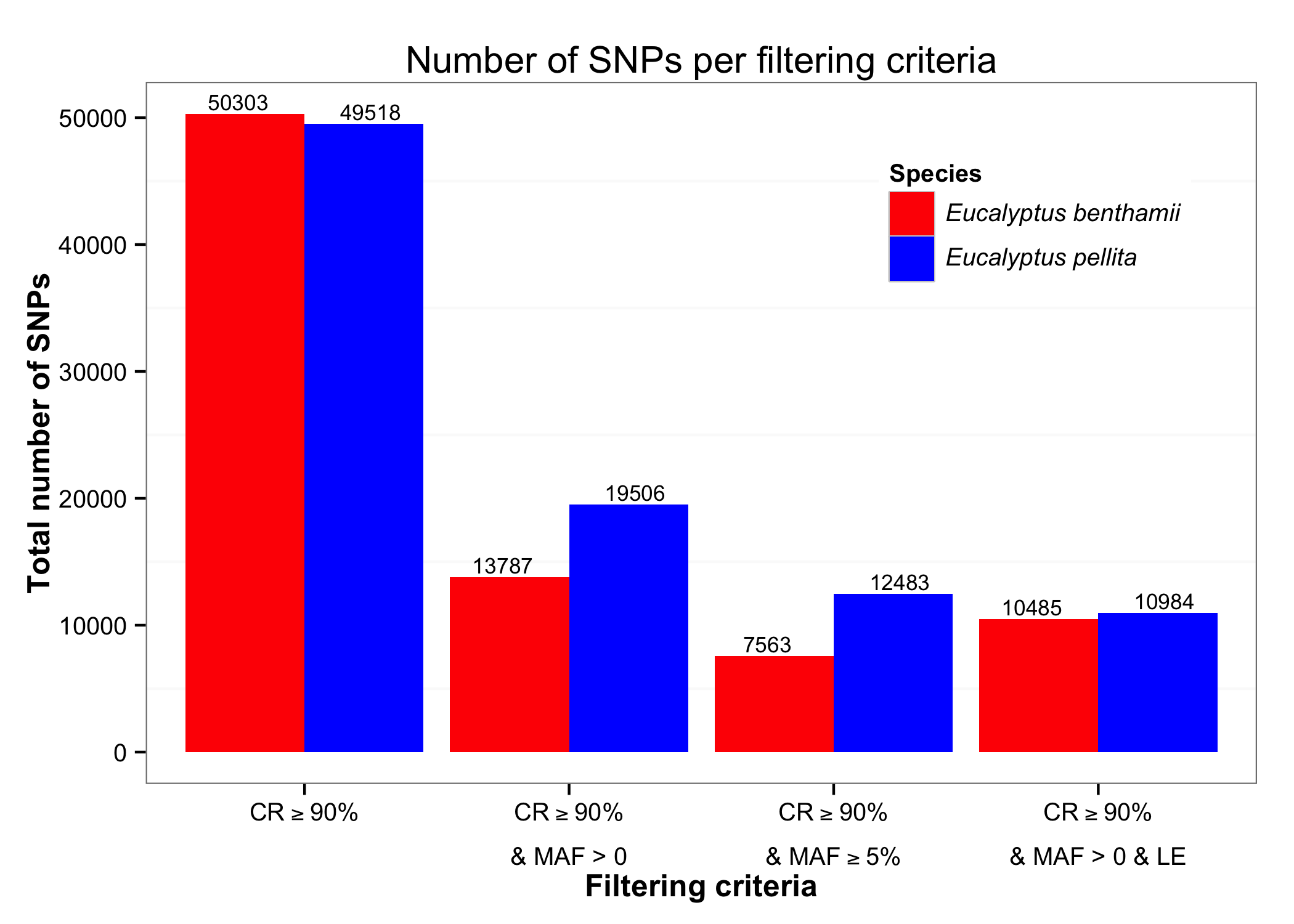

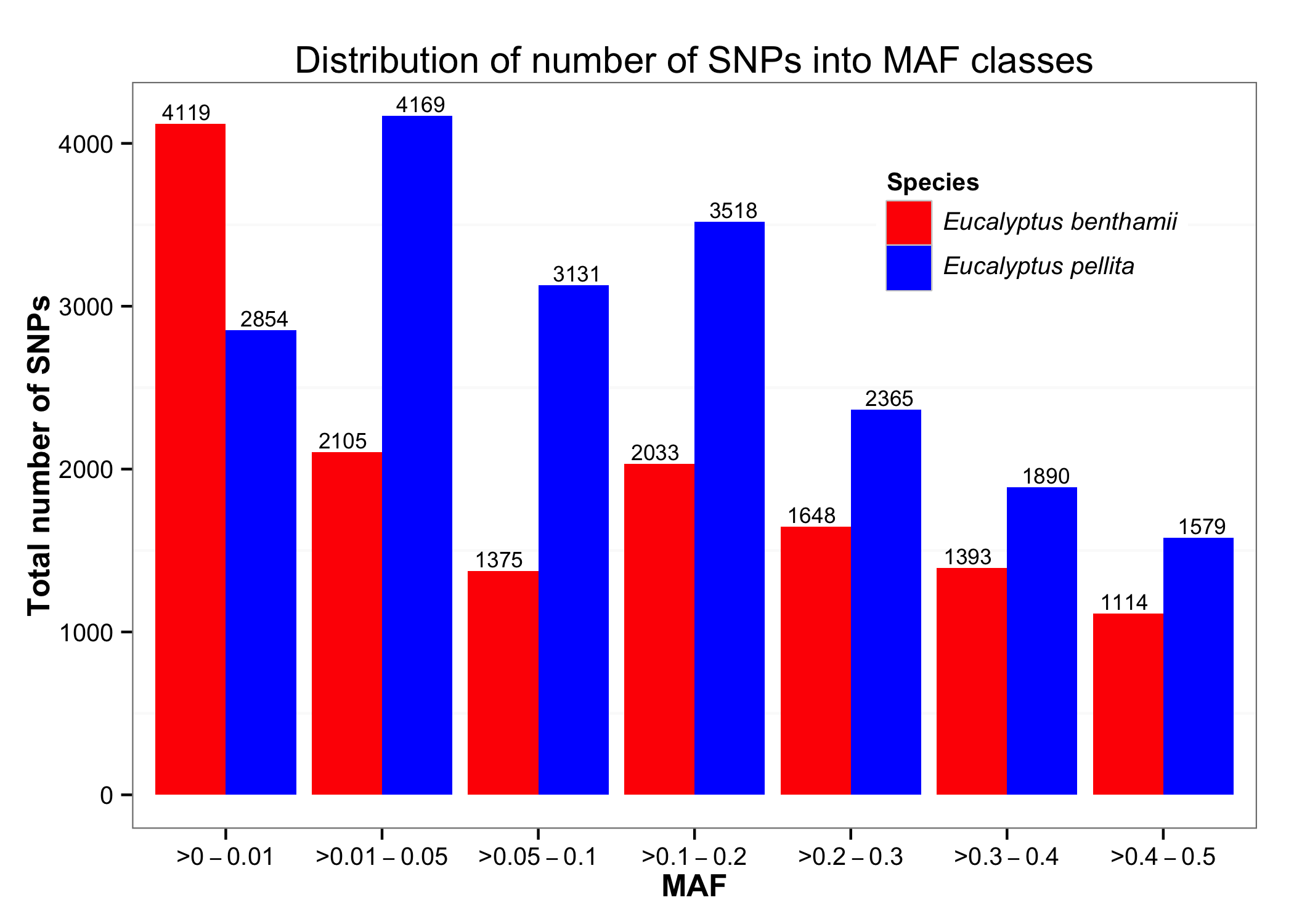


**A**

**B**

**Figure S1: Distribution of the numbers of SNPs for variable filtering criteria and MAFs classes.** (A) Distribution of the number of SNPs retained in variable filtering criteria, and (B) Distribution of the number of SNPs into MAF classes for *E. benthamii* and *E. pellita* for CR ≥ 90% and MAF > 0 (CR, Call Rate; MAF, Minimum Allele Frequency; LE, Linkage Equilibrium).

**
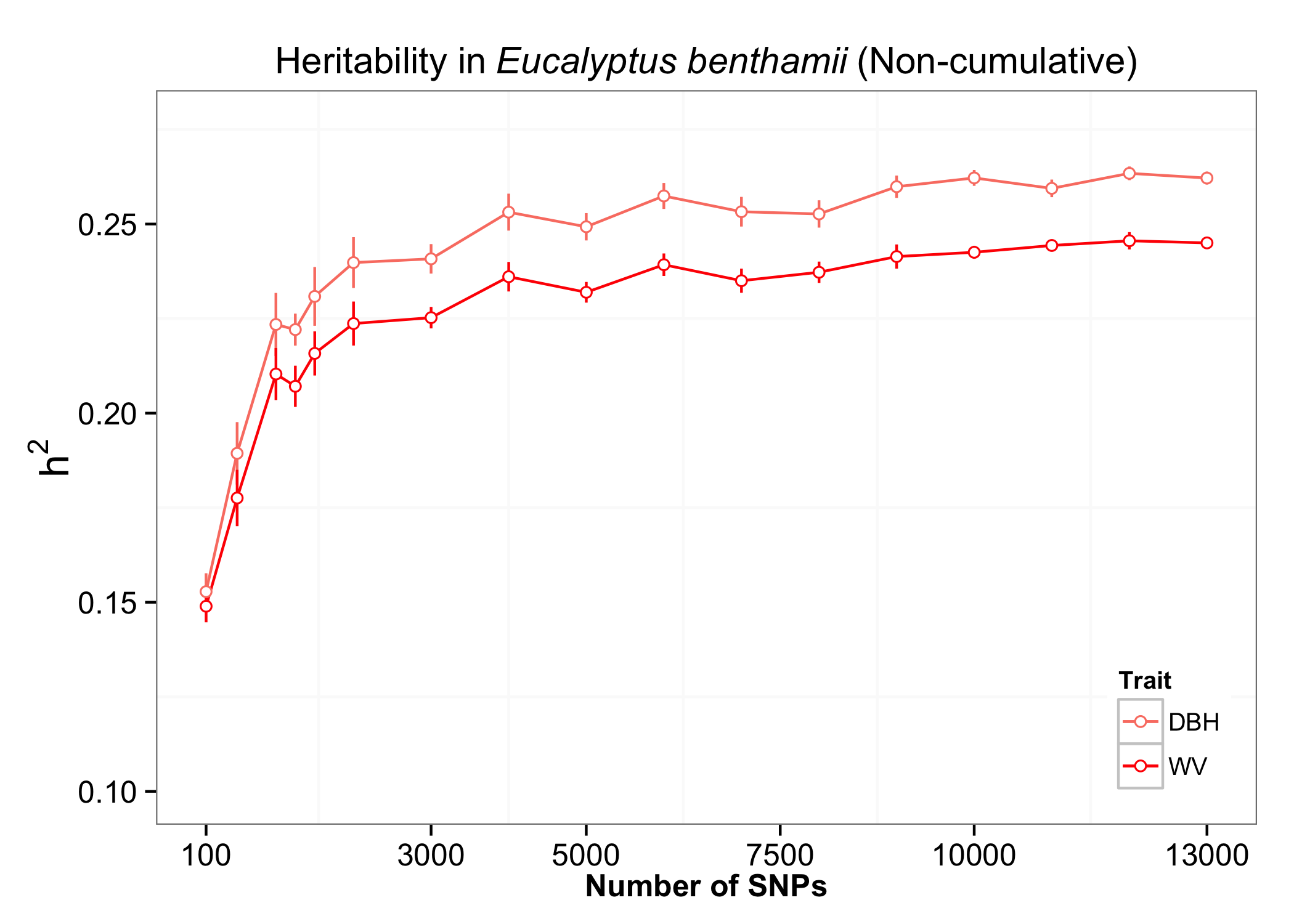

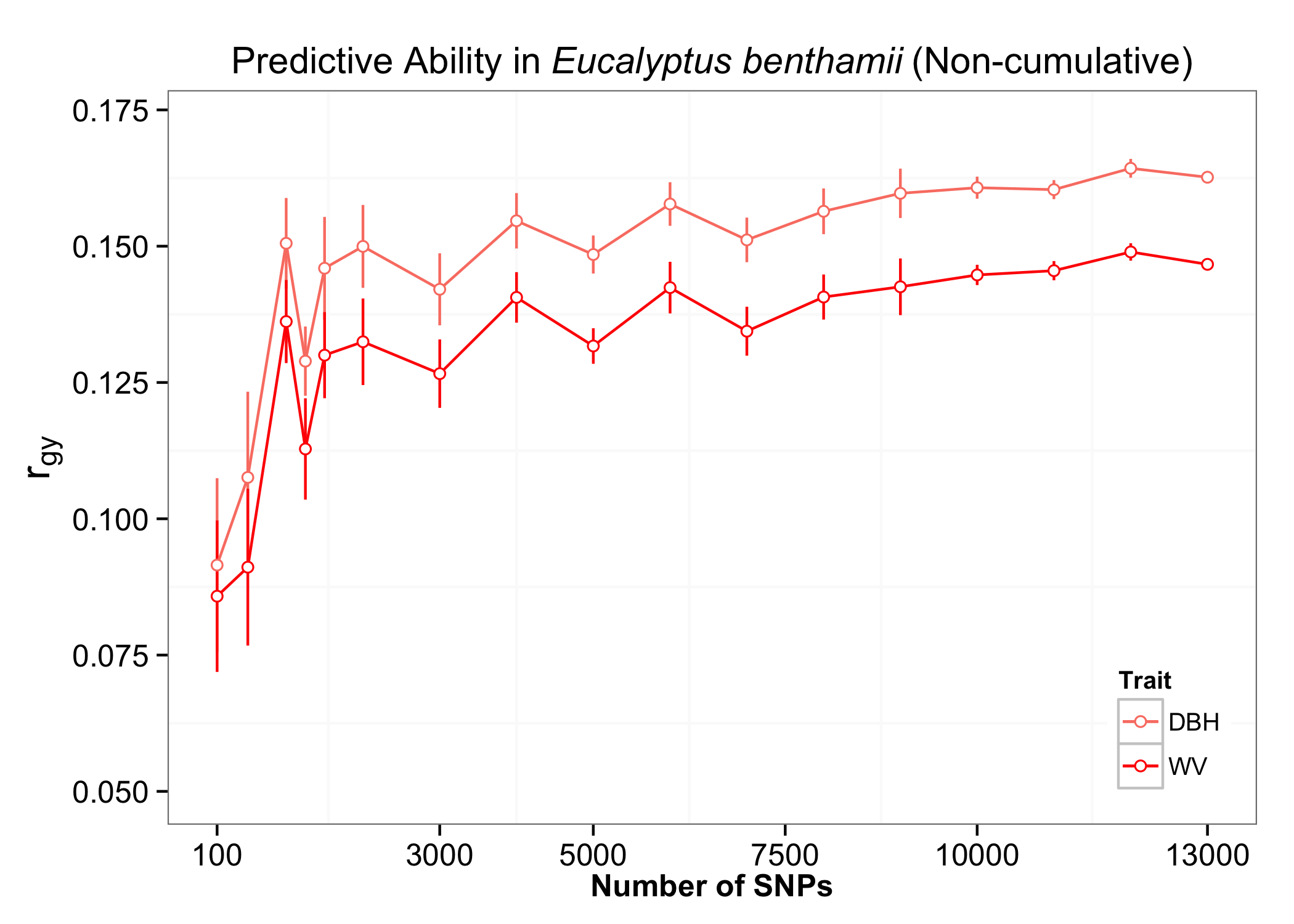
**

**C**

**D**

**A**

**B**

**
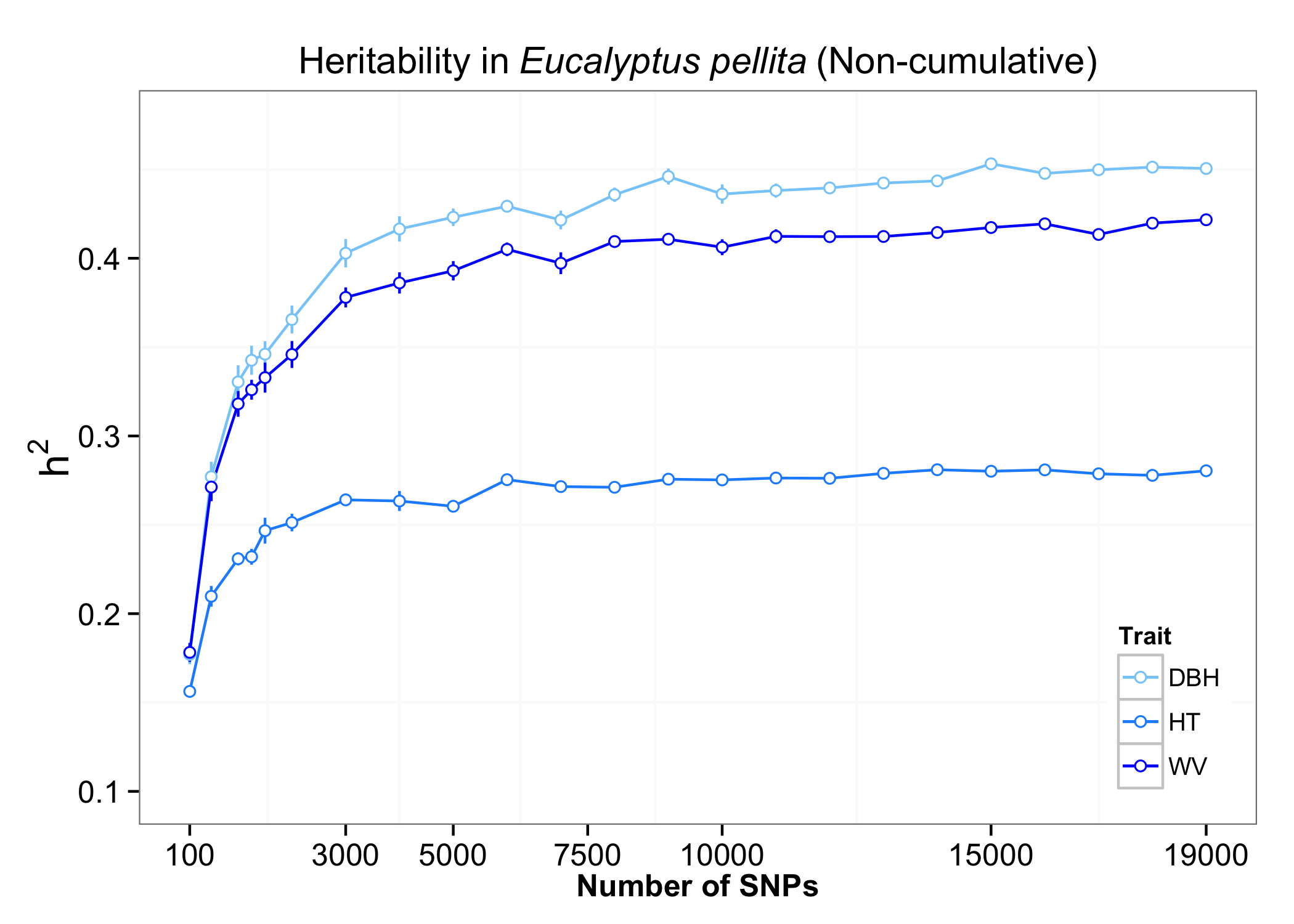

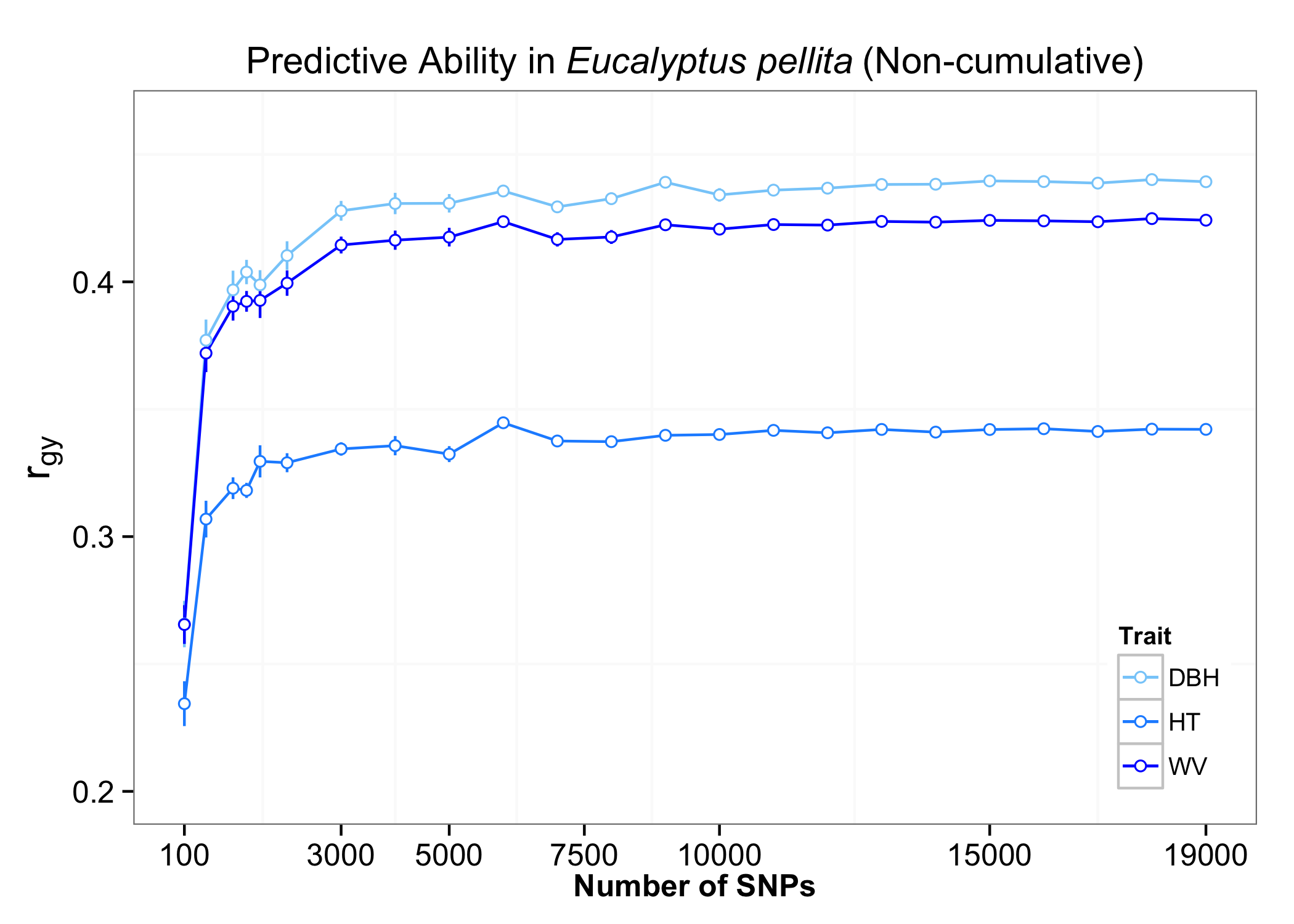
**

**Figure S2: Estimates of heritability (*h^2^*) and of predictive ability (*r_gy_*) with increasing numbers of SNPs for different traits using a non-cumulative approach to SNP sampling.** (A) and (B) estimates of *h^2^* and *r_gy_* for *E. benthamii*, respectively; (C) and (D) estimates of *h^2^* and *r_gy_* for *E. pellita*, respectively.


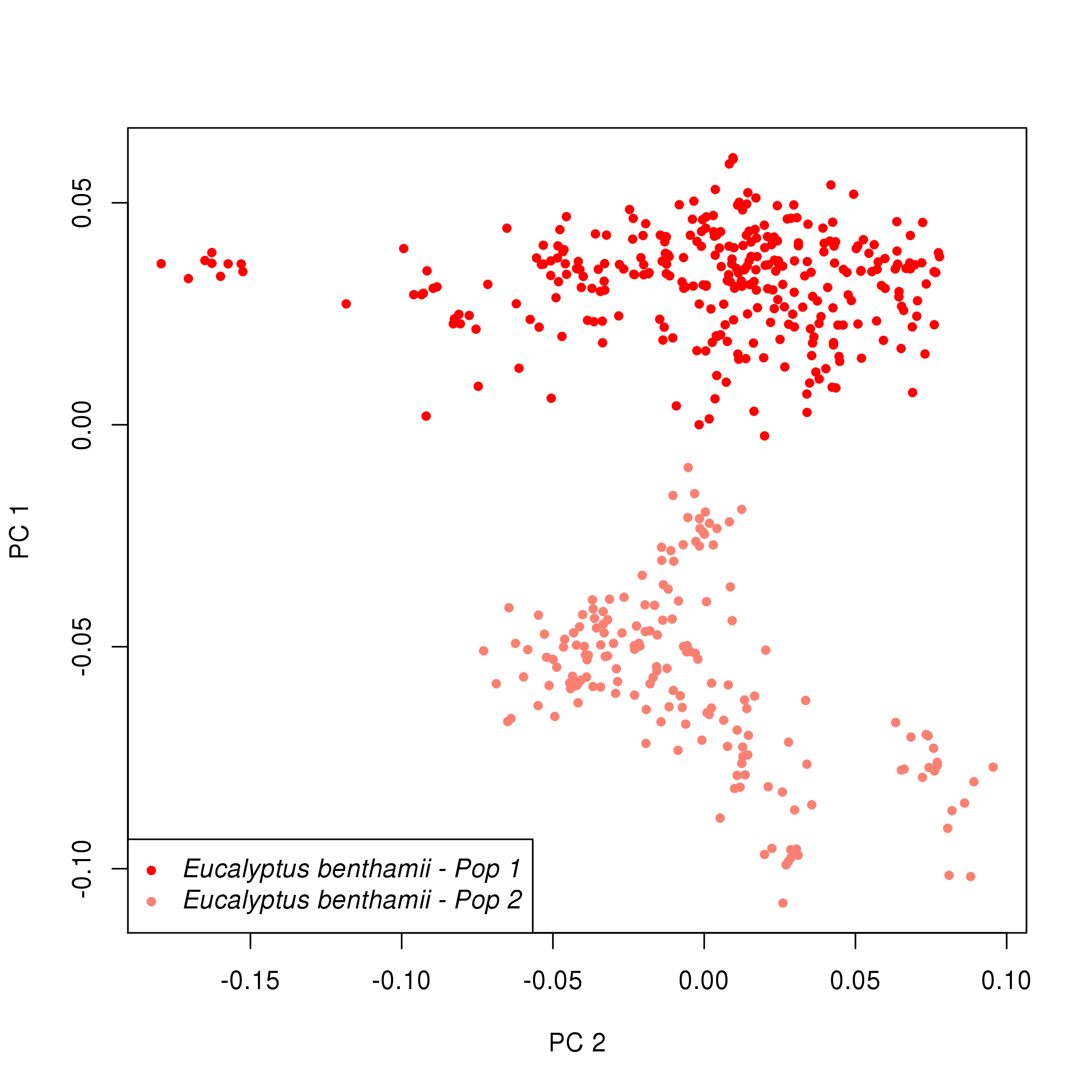

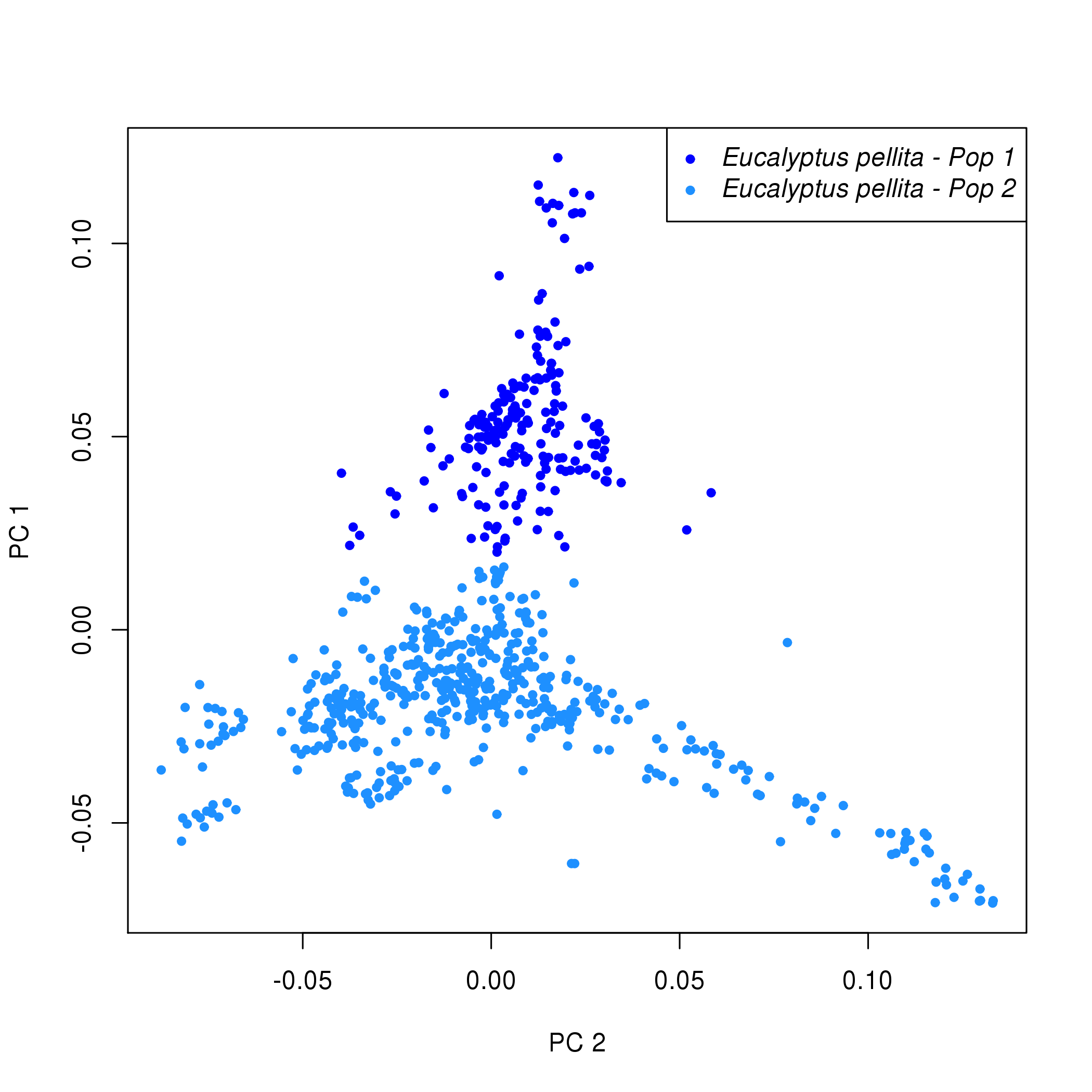


**B**

**A**

**Figure S3: Principal component analysis (PCA) of the 484 trees of *E. benthamii* (A) and 706 trees of *E. pellita* (B) used to split training and validation sets.** For *E. benthamii* 310 (red) and 174 (pink) individuals were used as training and validation sets. In *E. pellita*, the number of individuals used in each set were 192 (dark blue) and 514 (light blue).
